# A Sustainable Nanocomposite Au(Salen)@CC for Catalytic Degradation of Eosin Y and Chromotrope 2R Dyes

**DOI:** 10.1038/s41598-017-07707-6

**Published:** 2017-08-03

**Authors:** Vishal J. Mayani, Suranjana V. Mayani, Sang Wook Kim

**Affiliations:** Department of Advanced Materials Chemistry, College of Science and Technology, Dongguk University, Gyeongju, Gyeongbuk 780-714 Republic of Korea

## Abstract

Up to now, a very few catalysts have been developed approaching the heterogeneous catalytic degradation of Eosin Y and Chromotrope 2R dyes (Acid Red 29). The present study provides a complete perspective of recyclable nanocomposite **Au(Salen)@CC** for catalytic degradation of hazardous water pollutant dyes *viz*., Eosin Y & Chromotrope 2R using mild reaction conditions. New gold Salen complex doped carbon nanocomposite **Au(Salen)@CC** was developed by easy methodology using nano carbon cage (**CC**) prepared from low-priced Pyrolysis fuel oil (PFO) residue based Pitch. The UV-Vis adsorption spectroscopy results of Eosin Y and Chromotrope 2R dyes indicated complete degradation into acidic compounds which can be further mineralized to CO_2_ and H_2_O under mild reaction conditions. The heterogeneous catalyst recycled and reused successfully for four repeated experiments without loss in its adequate performance. This new sustainable and eco-friendly catalyst delivered significant degradation activity compared to existing reports and it can be further utilized for new multifunctional applications such as, radiopharmaceutical activities, heterogeneous catalysis and chiral resolution.

## Introduction

Dyes and pigments have ubiquitous presence in modern industries such as, textile, leather, food, paper and plastic industries. The discharged effluent of these factories is evidently contaminated and critically hazardous because of its adverse outcome on the environment and society. Countermeasure of this dye-associated waste has been greater field of exploration now a day. A red fluorescent dye from xanthene family, Eosin Y, has been normally utilized in several applications such as, semiconductor’s sensitizer^1^ biological stain, laser dye, fluorescent research, nanocomposite preparation and polymerization process^2^. Eosin Y has stability, resilience, and light absorptivity^3^. The extensive use of Eosin Y in sensitizing industries^3^ and as biological stain in biomedical research laboratories and for diagnostic intention^4^, cause carcinogenic exposure as dye waste due to its benzene and pyran rings as well as carboxyl group. Eosin Y affects surroundings by direct discharging into wastewater due to its toxic nature and its intense color. Chromotrope 2R (Acid Red 29) is one of the azo dyes consist of azo group (−N = N−), as chromophore group with aromatic structure having, −OH and –SO_3_H functional groups. Azo pigment also creates water contamination and serious environmental problem due to its carcinogenic, poisonous and harmful properties^5–9^.

Organic water contaminants are either moderately decomposed by using an oxidizing agent into biodegradable intermediates/important chemicals or complete mineralization into innocuous inorganic compounds such as CO_2_, H_2_O and inorganic salts, which remain in the aqueous phase. Further, the intermediates are broken down into simpler, easily treatable materials before they are released into the environment. The ultimate products usually reside of low molecular weight oxygenated molecules like acetic acid, propionic acid, ethanol etc^10^. Currently, a variety of techniques are being used for the removal or degradation of dyes from pollutant water, *e.g*. adsorption, coagulation, oxidation, reduction, microbiological, photo-catalysis and biotechnology. These techniques declined to remove or destroy pollutant completely, but it offered few drawbacks such as incomplete removal, high consumption of chemical reagent, expensive, slow and generation of toxic secondary contaminants^11^. Modified titanium dioxide (TiO_2_) is possibly most practiced photocatalyst for determining environmental issues specially wastewater purification^4, 12^. However, the TiO_2_ based photocatalysis experienced few fundamental technical hurdles that pauses its industrialization, *i.e*. limited utilization of visible light, small adsorption scope for hydrophobic pollutants, high aggregation tendency and recovery of the TiO_2_ composite after successive catalytic cycle^11^. The ability of biological treatment processes for decolorization of industrial effluents has limitations in that it is affected by the concentration, non-biodegradable toxic and inhibitory nature, alkalinity and elevated temperature of the dye effluents.

Salen Schiff base-transition metal complexes and their composites have been broadly researched due to its potential use as a promising catalyst in a wide range of organic transformations^13^. Hydrogen peroxide is a powerful and environment friendly oxidizing agents since the product of its degradation are H_2_O and O_2_^14^. Sodium borohydride (NaBH_4_) is widely used in catalytic reduction method of dyes^15^. Though activated carbon composite demonstrates its potential to remove the dyes, the high initial cost of carbon, coupled with problems associated with regeneration/reuse necessitates search for other developments. Owing to these reasons chemical oxidation-reduction methods are preferred. Various advanced oxidation/reduction processes of pollutant treatment have been modernized with low-cost, ecofriendlyness and high efficiency to reconcile the developing demand of direct wastewater treatment.

Very few reports have been published dealing with Eosin Y and Chromotrope 2R by catalytic degradation methods. Reusable, inexpensive and eco-friendly organic metal carbon nanocomposite have numerous supplementary combined features of organic moiety, metal and carbon base along with the potential for tuning the activity and selectively of nanocomposites. Combination of hetero atoms into porous carbon framework can engender active sites and most transition metals have been doped into porous nanocomposite. Transition metal nanocomposites have revealed huge importance over last decade because of their potential in applications as a solid catalyst, adsorbent, sensor, electrode, energy storage or its host^16^. To expand better awareness of the possibility of this treatment, herein, we report the development of new heterogeneous catalyst of Au(III) Salen complex onto carbon cage prepared from pyrolysis fuel oil based Pitch residue using a new and eco-friendly approach for the catalytic degradation of Eosin Y and Chromotrope 2R dyes. Gold Salen complex doped carbon nanocomposite **Au(Salen)@CC** has promise for the degradation of dyes under mild conditions (Eosin Y: room temperature (25 °C); Chromotrope 2R: 50 °C and atmospheric pressure) using environmentally friendly oxidant (H_2_O_2_) for Chromotrope 2R and reductant NaBH_4_ for Eosin Y. This sustainable catalyst has immense prospective for the application of cleaning organic dye polluted water and it can be further utilized for new multifunctional applications as well.

## Results

**Figure 1:**
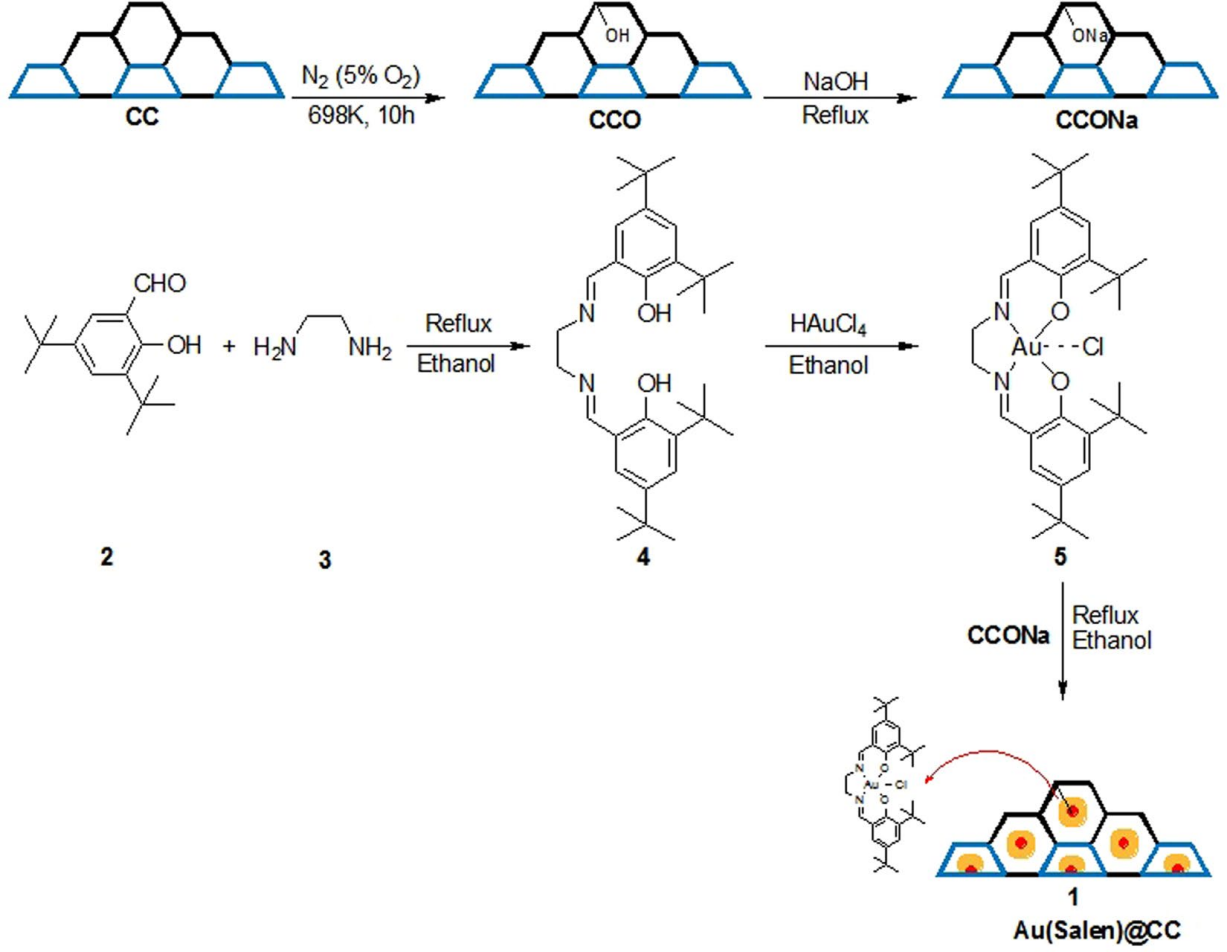
Synthesis route of carbon cage doped nanocomposite **Au(Salen)@CC 1**.

The organic-inorganic hybrid nanocomposite materials **CC**, **CCO**, **CCONa** and **Au(Salen)@CC** were synthesized by simple template method using nanosilicaball (**NSB**) followed by air oxidation and NaOH treatment and easy deposition of gold Salen complex on hierarchical **CC**, respectively (Fig. 1). All the composites and products were characterized by physico-chemical characterization methods.

### Powder X-ray diffraction analysis

**Figure 2:**
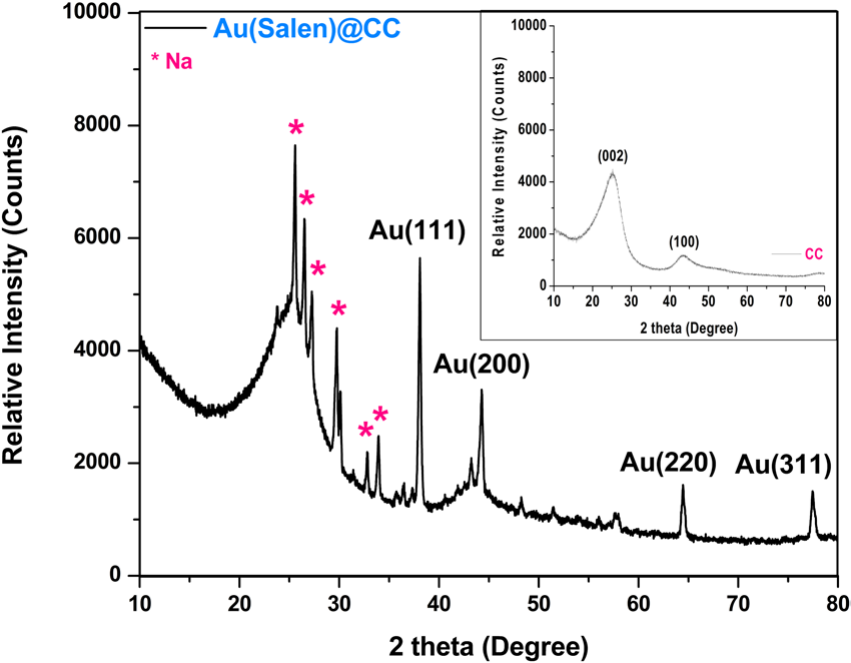
Wide-angle powder XRD patterns of **CC** and **Au(Salen)@CC**.

The wide angle XRD profile of **CC** showed two peaks with lower intensity corresponding to graphite type reflections from the (002) and (100) planes which were correspond to 2θ value 25.3° and 42.7°, respectively (Fig. 2), which were assigned to porous carbon^17–19^. Upon Salen complex of gold deposition in **CC**, XRD of the **Au(Salen)@CC** showed four new peaks at 2θ = 38.1°, 44.3°, 64.5° and 77.5°, which resembled to reflections of gold planes (111), (200) (220) and (311), respectively^19^. Some supplementary diffractions peaks of sodium metal planes observed at 2θ value 25.6°, 26.5°, 27.2°, 29.8°, 30.1°, 32.8° and 33.9°^18^. Though, the low-angle XRD of **CCO**, **CCONa**, **Au(Salen)@CC** did not show any characteristic peak (Fig. S5, supplementary information).

### Nitrogen adsorption–desorption study

The BET surface area of **NSB** and **CC** was 163 m^2^/g and 212 m^2^/g, respectively. **CC** was oxidized with a mixture of nitrogen and air (with 5% O_2_) to **CC**, the surface areas of **CCO** was 224 m^2^/g. The surface hydroxyl groups triggering and gold Salen complex incorporation to **CCO**, the surface area of **CCONa** and **Au(Salen)@CC** observed to 258 and 239 m^2^/g, respectively. An increase in the pore diameter from 71 to 162 Å was observed from **NSB** to **CC**. Upon oxidation and impregnation, the values decreased further for **CCO**: 16; **CCONa**: 16; and **Au(Salne)@CC**: 17 Å with respect to **CC**. The pore volume of **NSB** and **CC** was 0.290 cm^3^/g and 0.857 cm^3^/g, respectively. The pore volume of **CCO**, **CCONa** and **Au(Salen)@CC** also increased to 0.869, 1.003 and 1.002 cm^3^/g, respectively (Table S1 and Fig. S1, supplementary information).

### ^13^C CP-MAS NMR analysis

The presence of organic metal complex in **Au(Salen)@CC 1** was further monitored by solid state ^13^C CP-MAS NMR. It showed broad peaks in aromatic and aliphatic regions (58–189 δ ppm) (Fig. S4, supplementary information).

### TEM and SEM analysis

**Figure 3:**
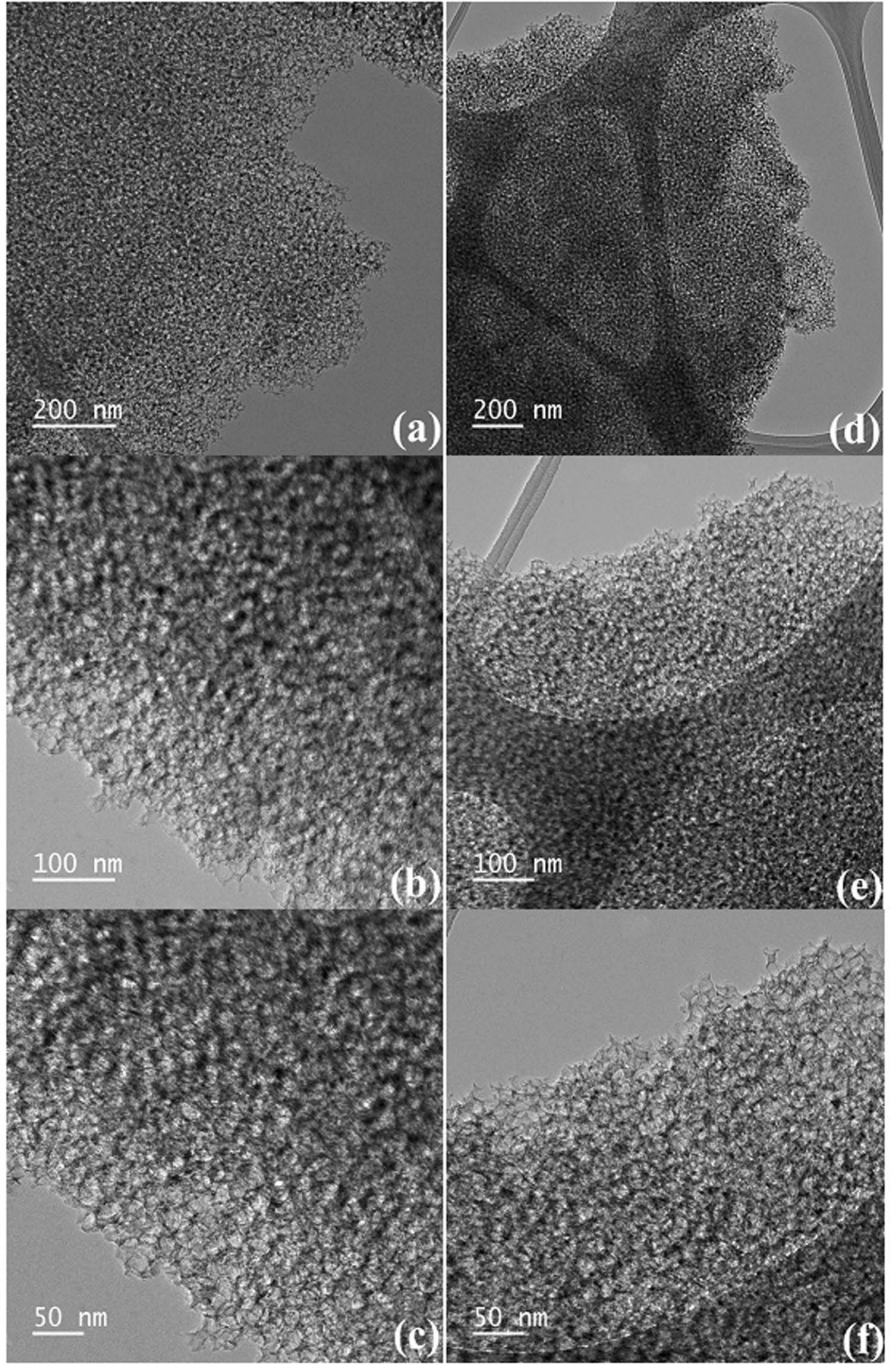
TEM images of **CCO** (**a–c**) and **Au(Salen)@CC** (**d–f**).

The TEM images of the **CCO**, **CCONa** and **Au(Salen)@CC** carbon materials are shown in Fig. 3. The Scanning electron microscopy image of the **CC**, **CCO**, **CCONa** and **Au(Salen)@CC** are shown in Fig. S7 (Supplementary information).

### Thermogravimetric analysis

TGA revealed minute weight losses of approximately 4.70 and 4.68% from 21 to 800 °C for **CCO** and **CCONa**, respectively. The loading of organic moiety in **Au(Salen)@CC** was found to be 13.95% as determined from the weight loss measured by thermo-gravimetric analysis carried out in the temperature range between 27 to 800 °C (Fig. S8, supplementary information).

### Catalytic activity study

**Figure 4:**
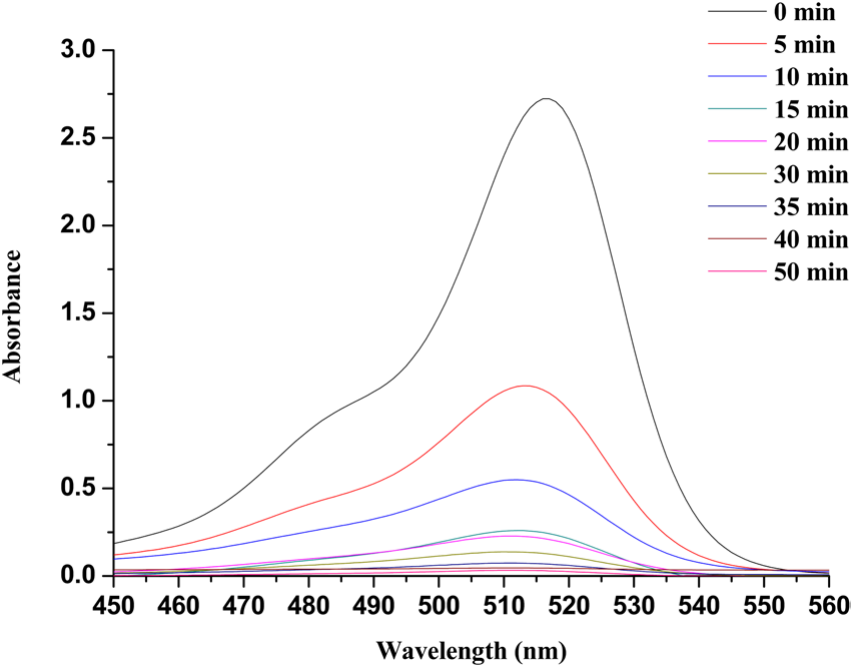
UV-vis absorption spectra of Eosin Y degradation with time catalyzed by **Au(Salen)@CC**.

**Figure 5:**
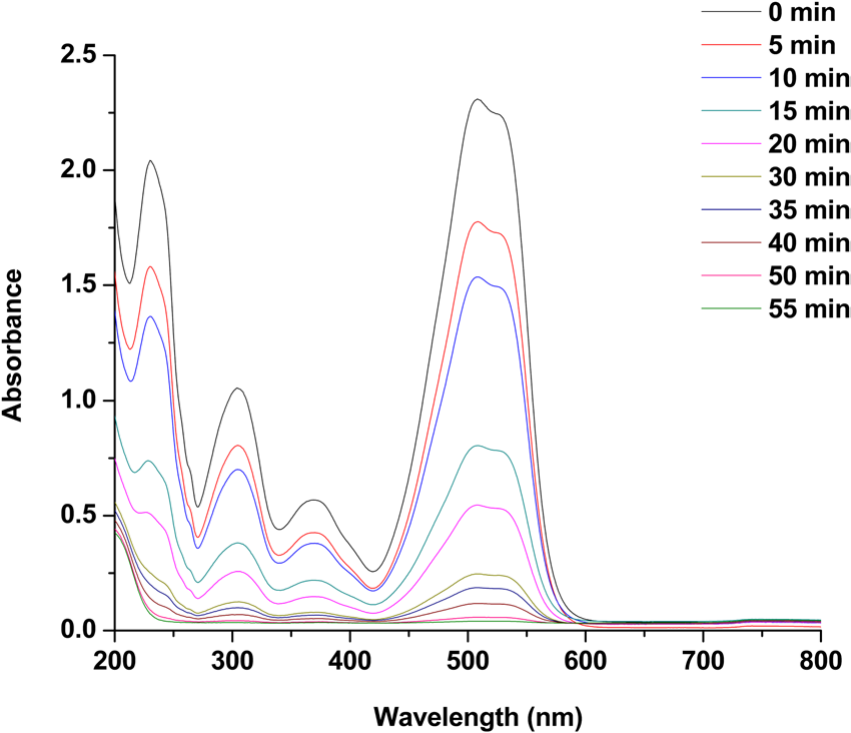
UV-vis absorption spectra of Chromotrope 2R degradation with time catalyzed by **Au(Salen)@CC**.

The Fig. 4 shows UV-vis spectra of Eosin Y degradation by **Au(Salen)@CC**. The spectrum of Eosin Y showed major absorbance band at 514 nm. It clearly indicates that in Eosin Y reduction in the presence of NaBH_4_, the intensity of absorbance peak became weaker as the reduction continued and totally disappeared after 50 min. We have also investigated oxidative degradation of Chromotrope 2R dye using Gold Salen complex doped carbon nanocomposite. The UV-vis measurement spectrum of chromotrope 2R is shown in Fig. 5. The spectrum of Chromotrope 2R exhibits major absorbance at 508 nm. When the catalyst was introduced to oxidation of Chromotrope 2R/H_2_O_2_ mixture, the reaction slowly loss in the absorbance of the dye was observed after 55 min.

## Discussion

The gold Salen complex doped carbon nanocomposite **Au(Salen)@CC** was developed by easy methodology using nano carbon cage (**CC**) prepared from low-priced pyrolysis fuel oil (**PFO**) residue based Pitch. All the composites and products were fully characterized by X-ray diffraction (XRD), ^13^C cross polarized magic angle spinning (CP-MAS) NMR, thermo-gravimetric analysis (TGA), N_2_ adsorption–desorption isotherm, scanning electron microscopy (SEM), transmission electron microscope (TEM), Gas chromatography mass spectrometry (GCMS), Liquid chromatography mass spectroscopy (LCMS) and Fourier transform-infrared (FT-IR). The detailed characterizations are given in supplementary information. UV-Vis spectroscopy was used for catalytic study of hazardous dyes and GCMS for identification of degraded products.

In powder X-ray diffraction analysis, the two broad peaks observed in the diffraction patterns of **CC** indicate that the crystallinity in the porous carbons is relatively small^19^. Four new peaks of **Au(Salen)@CC** designate the association of gold Salen complex verified the presence of gold complex in **CC** ^20^. Additional diffractions peaks supported the presence of sodium metal planes (JCPDS No. 011173 and 772082) are labeled in Fig. 2 ^21^. Metal incorporation has a considerable influence on the structural regularity of the **CC** materials^17, 22^. Recycled **Au(Salen)@CC** catalyst also shown identical XRD pattern after completion of catalytic cycle. It revealed that the heterogeneous catalyst **Au(Salen)@CC** was retained and stable under catalytic degradation condition (Fig. S8, supplementary information). In nitrogen adsorption study, the porosity of the carbons was generated by the release of small molecules during carbonization of the carbon precursors and the removal of the nano sized silica particles. Therefore, the different pore parameters of the immobilized **CC** and its precursors can be explained by the thermal stability of the **CC** and it remained intact after multiple treatment and immobilization (Table S1 and Fig. S1). ^13^C CP-MAS NMR analysis showed broad peaks in aromatic and aliphatic regions (58–189 δ ppm) corresponding to carbons originating from Salen complex immobilized on **CC** (Fig. S4, supplementary information). The presence of organic metal complex in **Au(Salen)@CC 1** was further confirmed by solid state ^13^C CP-MAS NMR. SEM image of **CC** has shown uniform hollow cores of hierarchically porous **CC** while images of **CCO**, **CCONa** and **Au(Salen)@CC** have shown well ordered nanoporous structure with the pores distributed consistently in the carbons. During the template synthesis process and thermal and chemical treatments, the hierarchically type framework of **CC** and **Au(Salen)@CC** is well-replicated and retained their well arranged hollow morphology (Fig. S7, supplementary information). Transmission electron microscopy image **CCO** has consistent nanoporous cores of hierarchically porous **CC**. The TEM images **CCONa** and **Au(Salen)@CC** have shown well retained nanoporous structure with the well pores distributed uniformly in the carbons upon thermo-chemical treatment (Fig. 3). Thermogravimetric analysis gives characteristic weight loss which shows the loading of organic moiety in **Au(Salen)@CC (**Fig. S8, supplementary information).

In Eosin Y degradation using **Au(Salen)@CC** as catalyst suggests that the benzene and pyran rings containing carboxyl group of Eosin Y destroyed and decomposed into simpler acid components which gradually will decay into its mineral components. GC-MS analysis could identify four products such as, 2-(2-formylphenyl)-2-carboxylate, 2-(2-3,6-dihydro-2H-pyran-4-yl)phenyl)2-carboxylate, 3,5-dibromocyclohex-5-ene-1,2,4-trione and (1Z,4E)-1,5-dibromo-3((Z)-2carboxyvinyl)-6-oxohexa-1,4-dien-2-olate. On the basis of degradation products, possible mechanism was suggested for catalytic degradation of Eosin Y (Figs S9 and S10, supplementary information)^4^. These four intermediate products undergo degradation to give malonic acid and oxalic acid which can be further mineralized to CO_2_ and H_2_O. As a result, **Au(Salen)@CC** can be used to decompose dye Eosin Y. Our research findings revealed that **Au(Salen)@CC** catalyst is more reliable and efficient compared to available literature reports^4^. We found 98.68% decomposition of Eosin Y within 50 min at normal pressure and room temperature, while recently practiced TiO_2_ based photocatalyst gave 96% degradation of Eosin Y under visible light irradiation for 180 min^4^. Decomposition reaction of Chromotrope 2R results into some active acid species due to degradation of aromatic structure containing azo group (−N = N−), −OH and –SO_3_H^9, 23^. GC-MS could identify oxalacetic acid, malonic acid, oxalic and oxamic acid as the final products that are mineralized to CO_2_ (Fig. S11, supplementary information)^24^. Equally, 98.78% degradation of Chromotrope 2R was achieved using **Au(Salen)@CC** catalyst within 55 min compared to report based on decomposition of the dye using Fenton process^9^. Our catalytic technique is simple, effective, and recyclable and requires very less time for complete degradation of dyes.

Besides, we have also conducted the catalyst optimization study by varying the amount of catalyst for the degradation of Eosin Y and Chromotrope 2R (Table 1). In case of both the dyes, when the catalyst load was increased from 1, 3, 5, 8, 10 g L^−1^ in different five experiments, the total conversation experienced reasonable enhancement. Required time for complete degradation of Eosin Y was 90, 70, 50, 50, and 50 min at catalyst amount 1, 3, 5, 8, 10 g L^−1^, respectively. Similarly, observed reaction completion time of Chromotrope 2R was 80, 70, 55, 55 and 55 min at catalyst load 1, 3, 5, 8, 10 g L^−1^, respectively. Thus, the optimum catalyst amount was found to be 5 g L^−1^, which produced maximum conversion in most favorable time of 50 and 55 min for Eosin Y and Chromotrope 2R, respectively. The UV-Vis spectra of catalyst optimization study were summarized Figs S12 to S21 in supplementary information.

**Table 1:** Effect of catalyst load on catalytic decomposition of Eosin Y and Chromotrope 2R.

Catalyst load (g L^−1^)	Eosin Y	Chromotrope 2R
	Reaction time (min)	Reaction time (min)
1	90	80
3	70	70
5	50	55
8	50	55
10	50	55

An attempt was made to recycle the catalyst, **Au(Salen)@CC**, after catalytic degradation of Eosin Y and Chromotrope 2R under optimized reaction conditions (Eosin Y: 2.7 × 10^−4^ mol L^−1^, 100 mL; NaBH_4_: 6.8 × 10^−4^ mol L^−1^, 0.025 g; Chromotrope 2R: 2.7 × 10^−4^ mol L^−1^, 50 mL; H_2_O_2_: 10^−4^ mol L^−1^, 50 mL; catalyst load: 5 gL^−1^; temperature: 25 °C (Eosin Y reaction) and 50 °C (Chromotrope 2R reaction). The aim was to evaluate the catalyst stability in degradation reaction, a basic necessity for its application at industrial scale. After the first catalytic reaction, the used **Au(Salen)@CC** catalyst was separated by filtration and the nanocomposite was thoroughly washed with distilled water. The resultant nanocomposite was dried overnight in vacuum desiccators at 100 °C and this revived **Au(Salen)@CC** catalyst was reused directly as a catalyst in the successive catalytic reaction. Four catalytic runs were carried out using the same catalyst with no observable loss of performance.

In conclusion, newly developed gold Salen doped carbon nanocomposite, **Au(Salen)@CC**, have potential functions in catalysis owing to their outstanding organic-inorganic hybrid physicochemical properties. The sustainable catalyst acts as a dynamic heterogeneous catalyst for catalytic decomposition of hazardous water pollutant dyes *viz*., Eosin Y and Chromotrope 2R. The complete degradation of Eosin Y and Chromotrope 2R dyes into acidic compounds was accomplished which can be further mineralized to CO_2_ and H_2_O under mild reaction conditions, it is more environmentally responsive and acceptable compared to earlier literature reports. The recyclability of eco-friendly catalyst is another advantage of the catalytic degradation process. The study is in progress towards the development of new applications such as energy storage and labeling of gold radioisotope for radiopharmaceutical activity.

## Methods

### Materials

Reagents and solvents such as ethanol, sodium hydroxide, hydrogen peroxide, Eosin Y (Dae-Jung Chemicals & Metals Co. Ltd., Korea), sodium borohydride, tetraethylorthosilicate, ethylenediamine (Aldrich, USA), hydrofluoric acid (J. T. Baker, USA), Chromotrope 2R, 3,5-di-tert-butyl-2-hydroxybenzaldehyde (Alfa Aesa, UK), and hydrogen tetrachloroaurate (III) hydrate (HAucl_4_) (Kojima Chemicals Co. Ltd, Japan) were employed as obtained. Petroleum residue pyrolysis fuel oil (**PFO**) was recieved from Yeocheon Naphtha Cracking Center (YNCC, South Korea). **PFO** based Pitch, Nano silica ball (**NSB**) and carbon cage (**CC**) were prepared according to the method reported previously using **PFO** based pitch residue^25^. All the solvents were purified by known method^26^.

### Characterization of materials

The characterization of **Au(Salen)@CC**, its precursors were fully accomplished by powder X–ray diffraction (PXRD, Phillips X’pert MPD diffractometer, Almelo, The Netherlands) in the low angle and high angle 2θ spectrum (0.5–10 and 10–80). ^1^H and ^13^C NMR were carried out by 500 and 125 MHz NMR spectrometer (Jeol, JNM-ECA500, Japan). Cross polarized magic angle spinning (CP-MAS) ^13^C NMR was used for the identification of **Au(Salen)@CC** catalyst (Agilent Unity Infinity Solid State 200 MHz NMR, USA). Fourier transform infrared spectroscopy (FT-IR, Perkin–Elmer Spectrometer, Massachusetts, USA) was conducted using a KBr pellet facility. Microanalysis of the compounds was accomplished by CHN analyzer (CE instruments, UK) and the percentage of gold associated with CC were measured by inductively coupled plasma optical emission spectroscopy (ICP-OES, JY Ultima 2CHR). The thermal strength of the carbon nanocomposite was discovered by thermogravimetric analysis (TGA, SDT600, TA instrument, USA). The BET surface area was performed by nitrogen adsorption-desorption data determined at 77 K adopting volumetric adsorption technique (Micromeritics ASAP-2010, USA). The micro-structural assessment of these nanocomposite were probed by scanning electron microscopy (SEM, LEO–1430, VP, UK) and transmission electron microscopy (TEM, JEM 2011, Jeol Corporation, Japan). The high performance liquid chromatography mass spectroscopy (LCMS, Agilent Technology, 6120 quadrupole LC/MS, USA) and gas chromatography mass spectroscopy (GCMS, HP-6890 MASS-5973, USA) were used for the identification synthesized compounds and degraded products. The complete degradation of Eosin-Y and Chromotrope-2R dyes was monitored by UV–vis spectroscopy (Varian Cary 4000, USA).

### Preparation of CCO and CCONa

The prepared petroleum pitch based carbon cage (**CC)** was oxidized with a mixture of nitrogen and air (with 5% O_2_) at 698 K for 10 h to obtain **CCO**. The surface hydroxyl groups of **CCO** were triggered by refluxing it with an aqueous solution of 0.1 N NaOH for an hour (Fig. 1). A sharp decrease in the pH value of the mixture from 13 to 10 was examined. This linking agent **CCONa** was washed with distilled water prior to constant pH = 8 and then dried at 393 K in an oven under vacuum^27^.

### Synthesis of Salen ligand 4

The Salen ligand used for the development of **Au(Salen)@CC** catalyst was synthesized by following procedure adapted from our group’s earlier report^28^. First, a mixture of 3,5-di-tert-butyl-2-hydroxybenzaldehyde **2** (23.40 g, 99.83 mmol) and ethylenediamine **3** (0.40 ml 5.9745 mmol) was refluxed for 6 h in 30 mL of ethanol (Fig. 1). The formation of compound **4** was observed by TLC analysis. The final solution was concentrated by rotary evaporator after cooling. The yellow color residue was washed with cool ethanol and dried in vacuum. Salen ligand **4** was purified by silica gel column chromatography. Yield 24.25 g (98%). ^1^H NMR (500 MHz, CDCl_3_): δ 13.63 [s, 2 H, *OH*], 8.38 [s, 2 H, NC*H*], 7.36 [d, *J* = 2.46, 2 H of Ar], 7.25 [CDCl_3_] 7.06 [d, *J* = 2.45, 2 H of Ar], 3.91 [s, 4 H, C*H*_*2*_], 1.43 [s, 18 H, CC*H*_*3*_] and 1.28 [d, *J* = 0.74, 18 H, CC*H*_*3*_] (Fig. S2). ^13^C NMR (125 MHz, CDCl_3_): δ 168 [*C* of Imine], 158 [*C* of Ar], 140 [*C* of Ar], 137 [*C* of Ar], 127 [*C* of Ar], 126 [*C* of Ar], 118 [*C* of Ar], 60 [*C*H_2_N], 35 [*C*CH_3_], 34 [*C*CH_3_], 32 [*C*H_3_] and 30 [*C*H_3_] (Fig. S3). Elemental analysis (Found) C: 77.84, H: 10.00, N: 5.65% (C/N = 13.78, C/H = 7.78). LCMS: *m/z* = 493.5 [M + H]^+^, 492.4 [M]^+^. FTIR (KBr): 2958, 2907, 2869, 2591, 1630, 1594, 1440, 1391, 1361, 1272, 1203, 1172, 1131, 1042, 974, 879, 849, 829, 773 and 710 cm^−1^.

### Synthesis of gold Salen complex 5

The gold (III) Salen complex was synthesized by refluxing an ethanolic solution of equimolar quantities of hydrogen tetrachloroaurate (III) hydrate (5 g in 50 mL, 14.71 mmol) and ligand **4** (7.25 g, 14.71 mmol, MW 492.37). The above mixture was refluxed for 4 h under steady stirring condition (Fig. 1). Finally, a large amount of pinkish gray solid was formed. The material was filtered and washed extensively with dry ethanol (3 × 5 mL) and dried air to afford the respective gold (III) Salen complex **5**. Yield 3.16 g (30%). LCMS: *m/z* = 723.4 [M]^+^. FTIR (KBr): 2978, 2912, 2802, 2744, 2710, 2676, 2573, 2519, 2418, 2279, 2056, 1601, 1487, 1344, 1085, 1034, and 820 cm^−1^.

### Preparation of gold Salen doped carbon nanocomposite Au(Salen)@CC

Functionalization of Salen complex **5** with treated carbon cage **CCONa** was achieved by following method. Activated **CCONa** (5.0 g) nanocomposite was refluxed with an ethanolic solution of 1.38 mmol of gold (III) Salen complex **5** (1.0 g) for 12 h (Fig. 1). The prepared nanocomposite was washed and purified by Soxhlet extraction process with dry ethanol for 8 h. Gold Salen complex immobilized on carbon cage was vacuum dried in an oven overnight at 110 °C. Yield: 5.903 gm. ^13^C CP-MAS NMR (12 KHz): δ 58–189 (broad peak, C of Salen complex). Elemental analysis (Found) C: 78.68, H: 1.19, N: 2.18%. Elemental analysis and ICP results showed that **Au(Salen)@CC** contained > 78 wt% carbon and 2.99 wt% Au after compound **5** immobilization on **CC**, respectively. Solid reflectance UV-Vis: 220, 280, 340 nm. FTIR (KBr): 3450, 2917, 2803, 2677, 2347, 2058, 1600, 1502, 1086 and 1034 cm^−1^.

### Catalytic reduction of Eosin Y and oxidation of Chromotrope 2R dyes

Catalytic degradation of Eosin Y and Chromotrope 2R dyes was executed in a glass reactor attached with a condenser. The reduction reaction of Eosin Y was carried out under typical conditions, by combining Eosin Y (100 mL, 2.7 × 10^−4 ^mol L^−1^), NaBH_4_ (0.025 g, 6.8 × 10^−4 ^mol L^−1^) with the catalyst **Au(Salen)@CC** (5 g L^−1^). The reaction mixture was stirred with 180 rpm rate for 50 min at room temperature (25 °C) and normal pressure. The oxidation reaction of Chromotrope 2R was carried out with 50 mL Chromotrope 2R (2.7 × 10^−4 ^mol L^−1^), 50 mL H_2_O_2_ (10^−4 ^mol L^−1^) using 5 g L^−1^ **Au(Salen)@CC** catalyst for 55 min at 50 °C temperature, normal pressure, and 180 rpm rate. After the completion of reaction, the catalyst, **Au(Salen)@CC**, was separated by centrifugation. The complete degradation of Eosin Y and Chromotrope 2R was monitored using UV–visible spectroscopy (at maximum adsorption wavelength of 514 nm and 508 nm for Eosin Y and Chromotrope 2R, respectively. GCMS was used for product identification. After each reaction run was completed, the products were extracted with chloroform by mixing equal volumes of the product mixture and chloroform. The solution was sonicated for several minutes allowed to settle down layers. 0.6 μL of the extracted layer of the mixures were investigated by GC-MS. In GC-MS, the temperature of injection was 250 °C. The starting temperature was preserved at 50 °C for 5 min and then raised to 180 °C at 10 °C min^−1^ rate, retained at present temperature for 5 min, then increased to 250 °C at 10 °C min^−1^ rate, and again retained at this temperature for 5 min.

## Electronic supplementary material

Supplementary Information
