# Supplementary material for: A Sustainable Nanocomposite Au(Salen)@CC for Catalytic Degradation of Eosin Y and Chromotrope 2R Dyes
Source: Sci Rep. 2017 Aug 3;7:7239. doi: 10.1038/s41598-017-07707-6 (PMC5543062; doi:10.1038/s41598-017-07707-6)
Supplement: Supplementary file 1 — Supplementary Information [file 41598_2017_7707_MOESM1_ESM.pdf]

Supplementary Information

A sustainable nanocomposite Au(Salen)@CC for catalytic degradation of Eosin Y and Chromotrope 2R dyes

Vishal J. Mayani, Suranjana V. Mayani, Sang Wook Kim*

Department of Advanced Materials Chemistry, College of Science and Technology, Dongguk University, Gyeongju, Gyeongbuk 780-714, Republic of Korea, Email: swkim@dongguk.ac.kr, Tel: +82-54-770-2216, Fax: +82-54-770-2386

The characterization of **Au(Salen)@CC,** its precursors were fully accomplished by powder X–ray diffraction (PXRD, Phillips X′pert MPD diffractometer, Almelo, The Netherlands) in the low angle and high angle 2θ spectrum (0.5-10 and 10–80). ^{1}H and ^{13}C NMR were carried out by 500 and 125 MHz NMR spectrometer (Jeol, JNM-ECA500, Japan). Cross polarized magic angle spinning (CP-MAS) ^{13}C NMR was used for the identification of **Au(Salen)@CC** catalyst (Agilent Unity Infinity Solid State 200 MHz NMR, USA). Fourier transform infrared spectroscopy (FT-IR, Perkin–Elmer Spectrometer, Massachusetts, USA) was conducted using a KBr pellet faclility. Microanalysis of the compounds was accomplished by CHN analyzer (CE instruments, UK) and the percentage of gold associated with CC were measured by inductively coupled plasma optical emission spectroscopy (ICP-OES, JY Ultima 2CHR). The thermal strength of the carbon nanocomposite was discovered by thermogravimetric analysis (TGA, SDT600, TA instrument, USA). The BET surface area was performed by nitrogen adsorption-desorption data determined at 77 K adopting volumetric adsorption technique (Micromeritics ASAP-2010, USA). The micro-structural assessment of these nanocomposite were probed by scanning electron microscopy (SEM, LEO–1430, VP, UK) and transmission electron microscopy

(TEM, JEM 2011, Jeol Corporation, Japan). The high performance liquid chromatography mass spectroscopy (LCMS, Agilent Technology, 6120 quadrupole LC/MS, USA) and gas chromatography mass spectroscopy (GCMS, HP-6890 MASS-5973, USA) were used for the identification synthesized compounds and degraded products. The complete degradation of Eosin-Y and Chromotrope-2R dyes was monitored by UV−vis spectroscopy (Varian Cary 4000, USA). The detailed characterizations are given in below.

Nitrogen adsorption−desorption study

Table S1 lists the BET surface area, BJH pore diameter, and total pore volumes. The BET surface area of **NSB** and **CC** was 163 m^2/g and 212 m^2/g, respectively. **CC** was oxidized with a mixture of nitrogen and air (with 5% O_2) to **CC**, the surface areas of CCO was 224 m^2/g. The surface hydroxyl groups triggering and gold Salen complex incorporation to CCO, the surface area of **CCONa** and **Au(Salen)@CC** observed to 258 and 239 m^2/g, respectively. An increase in the pore diameter from 71 to 162 Å was observed from **NSB** to **CC**. Upon oxidation and impregnation, the values decreased further for **CCO**: 16; **CCONa**: 16; and **Au(Salne)@CC**: 17 Å with respect to **CC**. The pore volume of NSB and CC was 0.290 cm^3/g and 0.857 cm^3/g, respectively. The pore volume of **CCO**, **CCONa** and **Au(Salen)@CC** also increased to 0.869, 1.003 and 1.002 cm^3/g, respectively. The porosity of the carbons was generated by the release of small molecules during carbonization of the carbon precursors and the removal of the nano sized silica particles. Therefore, the different pore parameters of the carbons can be explained by the thermal stability of the carbon precursor (Figure S1).

FT-IR Analysis Data:

CCO: FTIR (KBr): 3441, 2348, 2326, 1708, 1580, 1356 and 1220 cm^{-1}. **CCONa**: FTIR (KBr): 3437, 2922, 2362, 2343, 1736, 1578 and 1232 cm^{-1}. **Ligand 4**: FTIR (KBr): 2958, 2907, 2869, 2591, 1630, 1594, 1440, 1391, 1361, 1272, 1203, 1172, 1131, 1042, 974, 879, 849, 829, 773 and 710 cm^{-1}. **Complex 5:** FTIR (KBr): 2978, 2912, 2802, 2744, 2710, 2676, 2573, 2519, 2418, 2279, 2056, 1601, 1487, 1344, 1085, 1034, and 820 cm^{-1}. **Au(Salen)@CC**: FTIR (KBr): 3450, 2917, 2803, 2677, 2347, 2058, 1600, 1502, 1086 and 1034 cm^{-1}. Recycled **Au(Salen)@CC**: FTIR (KBr): 3461, 3391, 2354, 1939, 1608, 1168, 1031, 780 cm^{-1}.

Recycled Catalyst Au(Salen)@CC data:

FTIR (KBr): 3461, 3391, 2354, 1939, 1608, 1168, 1031, 780 cm^{-1}.

Elemental analysis (Found) C: 78.85, H: 1.59, N: 2.03%.

Elemental analysis and ICP results showed that **Au(Salen)@CC** contained > 78 wt% carbon and 2.18 wt% Au after recycling the catalyst.

Decomposition conversion calculation:

The percentage decomposition conversion of Eosin Y and Chromotrope 2R are calculated according to the relation:

$$\text{Conversion (\%)} = [(C_0 - C_t)/ C_0] \times 100$$

Where C_0 is the initial concentration (mol L^{-1}), C_t the concentration at any time after the reaction starts, t the time (min).

Table S1.

Physico-chemical data of **NSB**, **CC**, **CCO**, **CCONa** and **Au(Salen)@CC**.

Sr. No.	Compound	BET Surface Area (m^2/g)	Total Pore Volume (cm^3/g)	BJH Pore Diameter (Å)	Langmuir Surface Area (m^2/g)
1.	**NSB**	163	0.290	71	219
2.	**CC**	212	0.857	162	271
3.	**CCO**	224	0.869	16	299
4.	**CCONa**	258	1.003	16	365
5.	**Au(Salen)@CC**	239	1.002	17	340

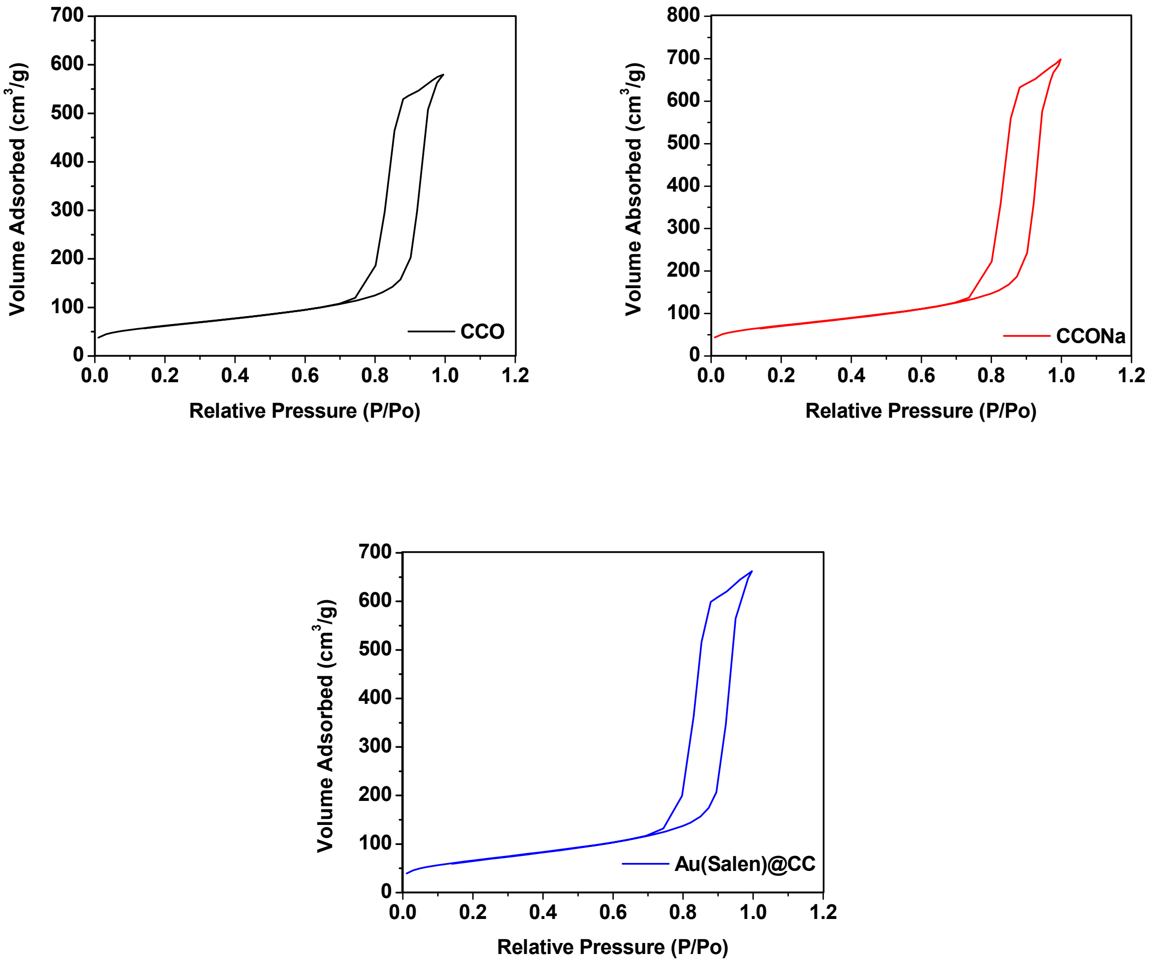

Figure S1. Nitrogen adsorption-desorption isotherms of **CCO, CCONa** and **Au(Salen)@CC**

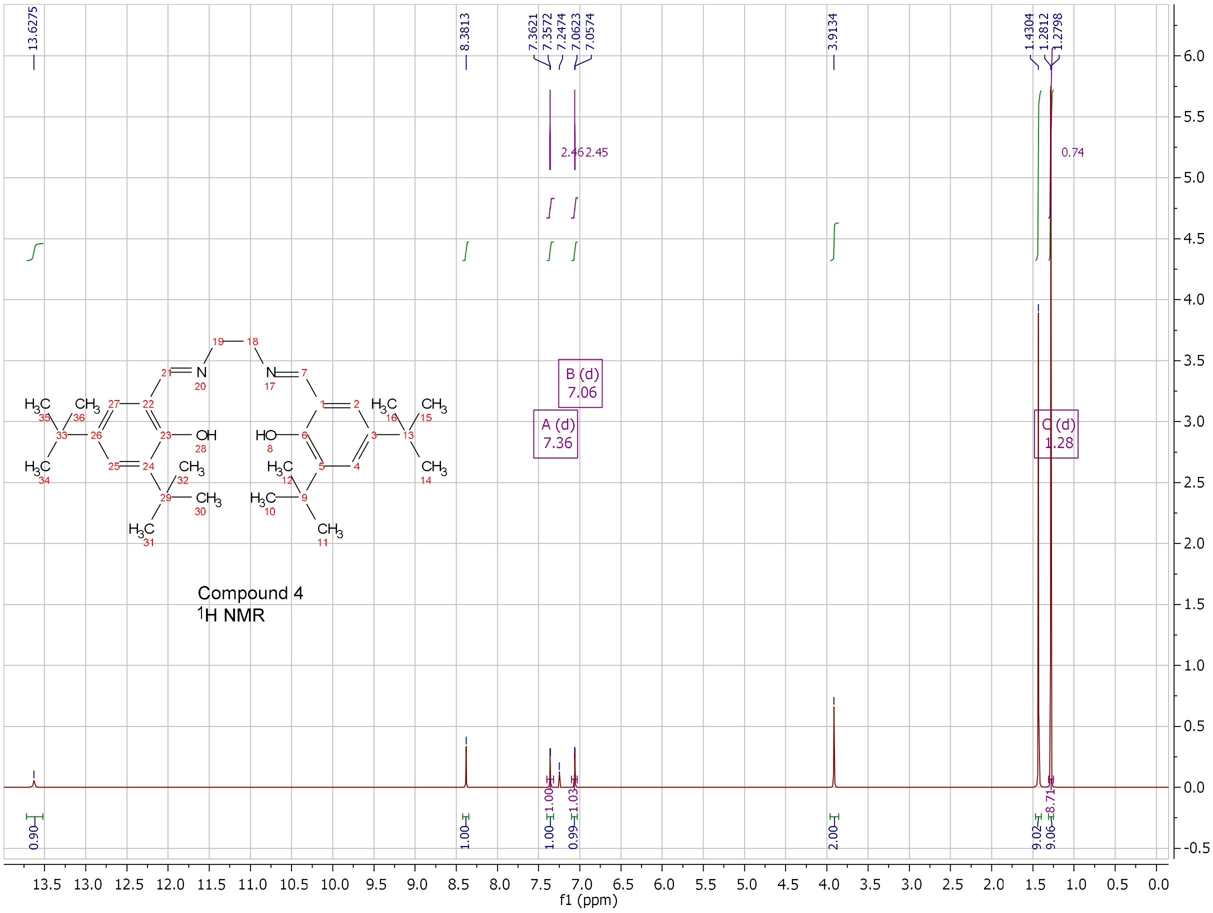

Figure S2. ^{1}H NMR of Compound **4**

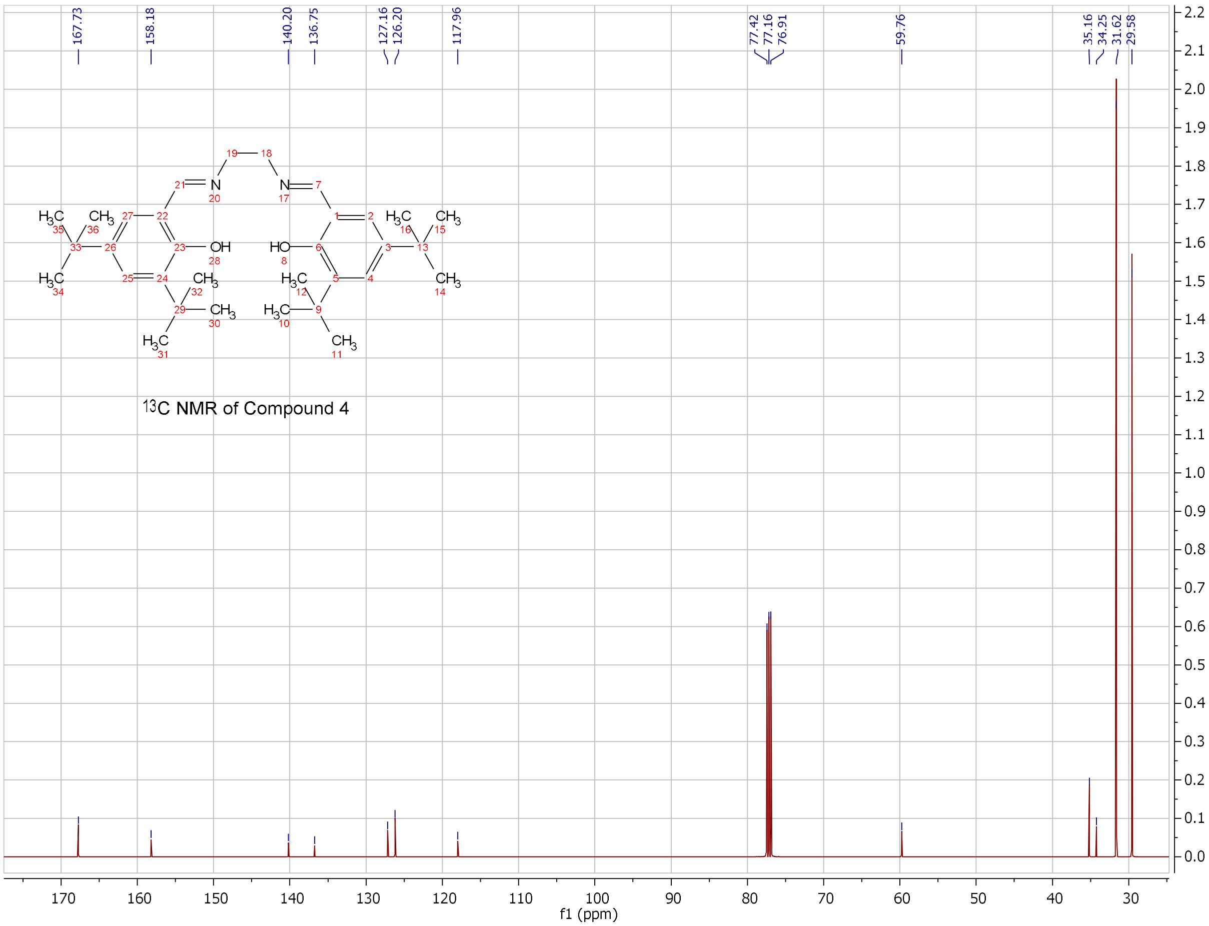

Figure S3. ^{13}C NMR of Compound **4**

^{13}C CP-MAS NMR Analysis

The presence of organic metal complex in **1** was further confirmed by solid state ^{13}C CP-MAS NMR that showed broad peaks in aromatic and aliphatic regions (58−189 δ ppm) corresponding to carbons originating from Salen complex immobilized on CC (Fig. S4).

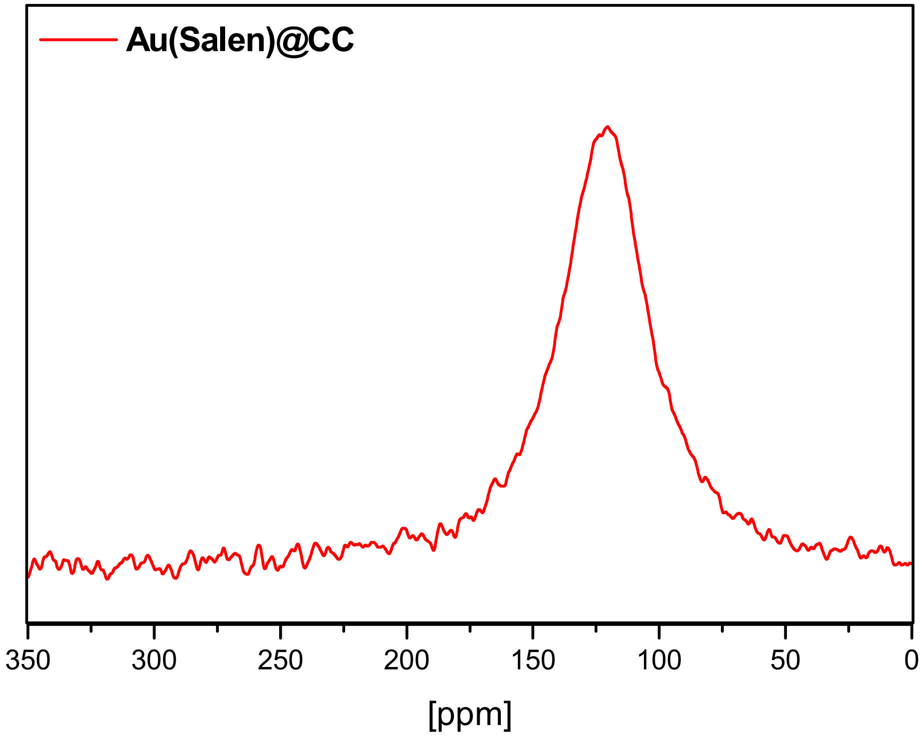

Figure S4. Solid State ^{13}C CP-MAS NMR spectra of **Au(Salen)@CC**.

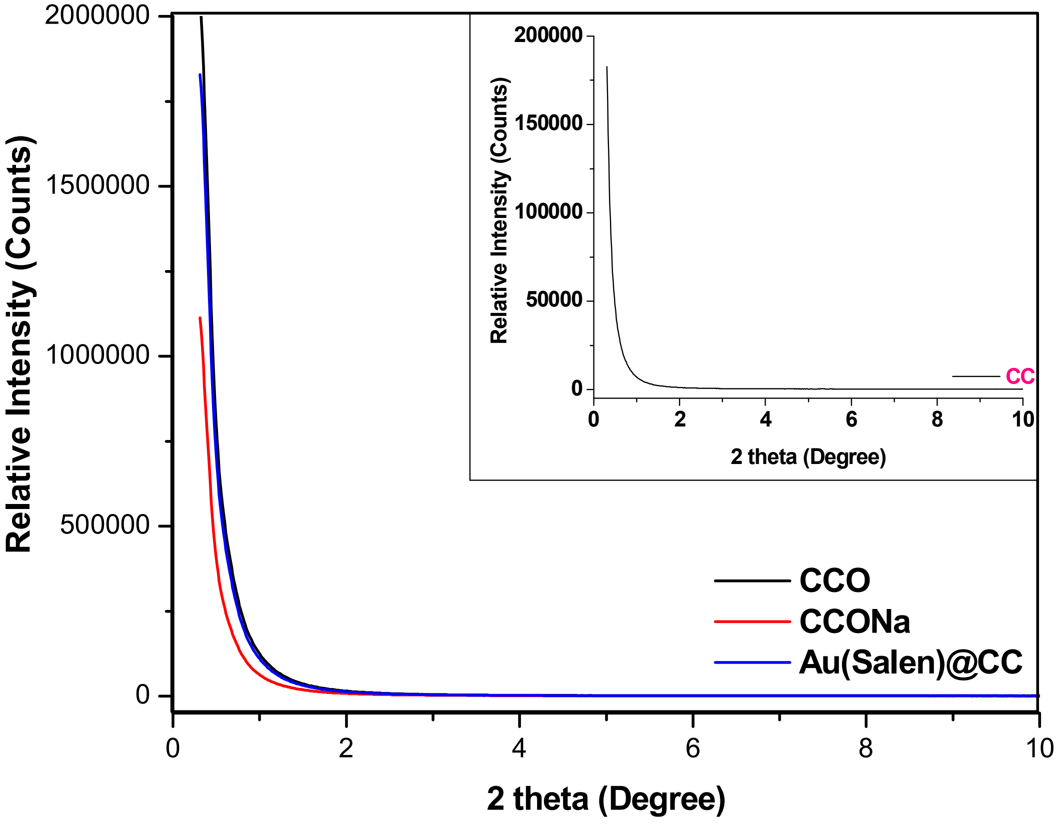

Figure S5. Low-angle powder XRD patterns of **CC, CCO, CCONa** and **Au(Salen@CC**.

Solid reflectance UV-Vis analysis

The solid reflection UV-Vis spectra of the immobilized **Au(Salen)@CC** shows ligand charge transfer band at 220, 280 and 340 nm confirming the presence of gold Salen complex of carbon cage (Fig. S6).

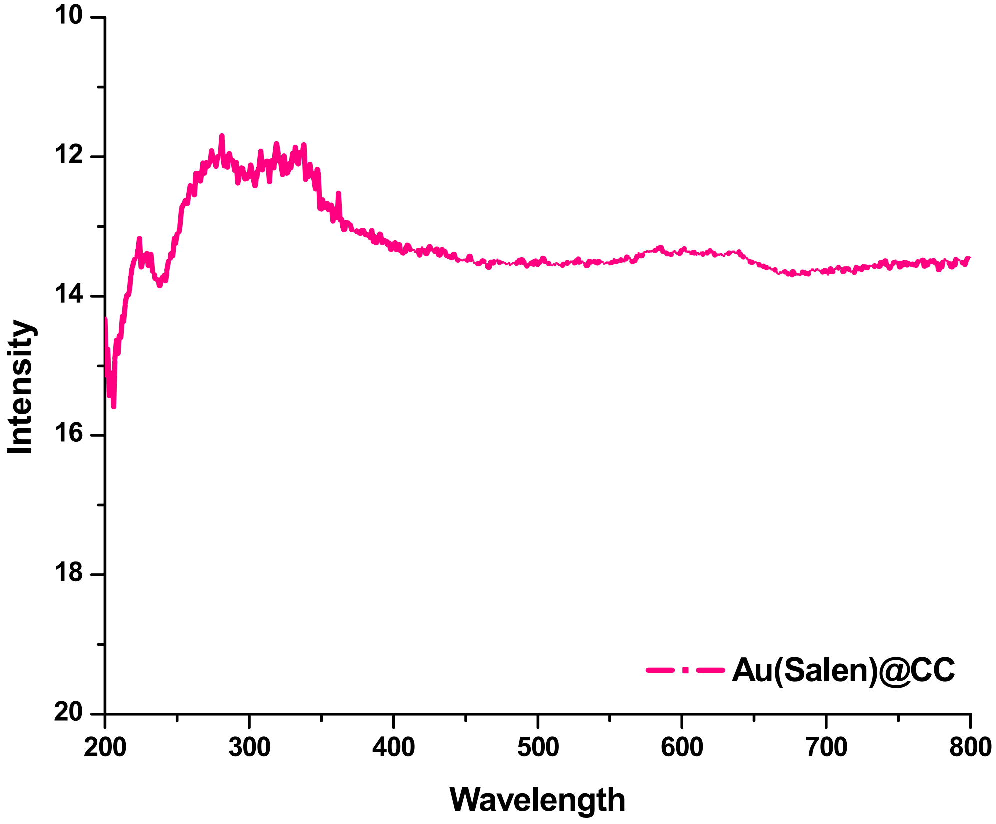

Figure S6. Solid reflectance UV-vis spectra of **Au(Salen@CC**.

Scanning electron microscopy analysis

The SEM images of the **CC**, **CCO**, **CCONa** and **Au(Salen)@CC** are shown in Fig. S7. Scanning electron microscopy image CC has shown uniform hollow cores of hierarchically porous CC while images of **CCO**, **CCONa** and **Au(Salen)@CC** have shown well ordered nanoporous structure with the pores distributed consistently in the carbons. During the template synthesis process and thermal and chemical treatments, the hierarchically type framework of **CC** and **Au(Salen)@CC** is well-replicated and retained their well arranged hollow morphology.

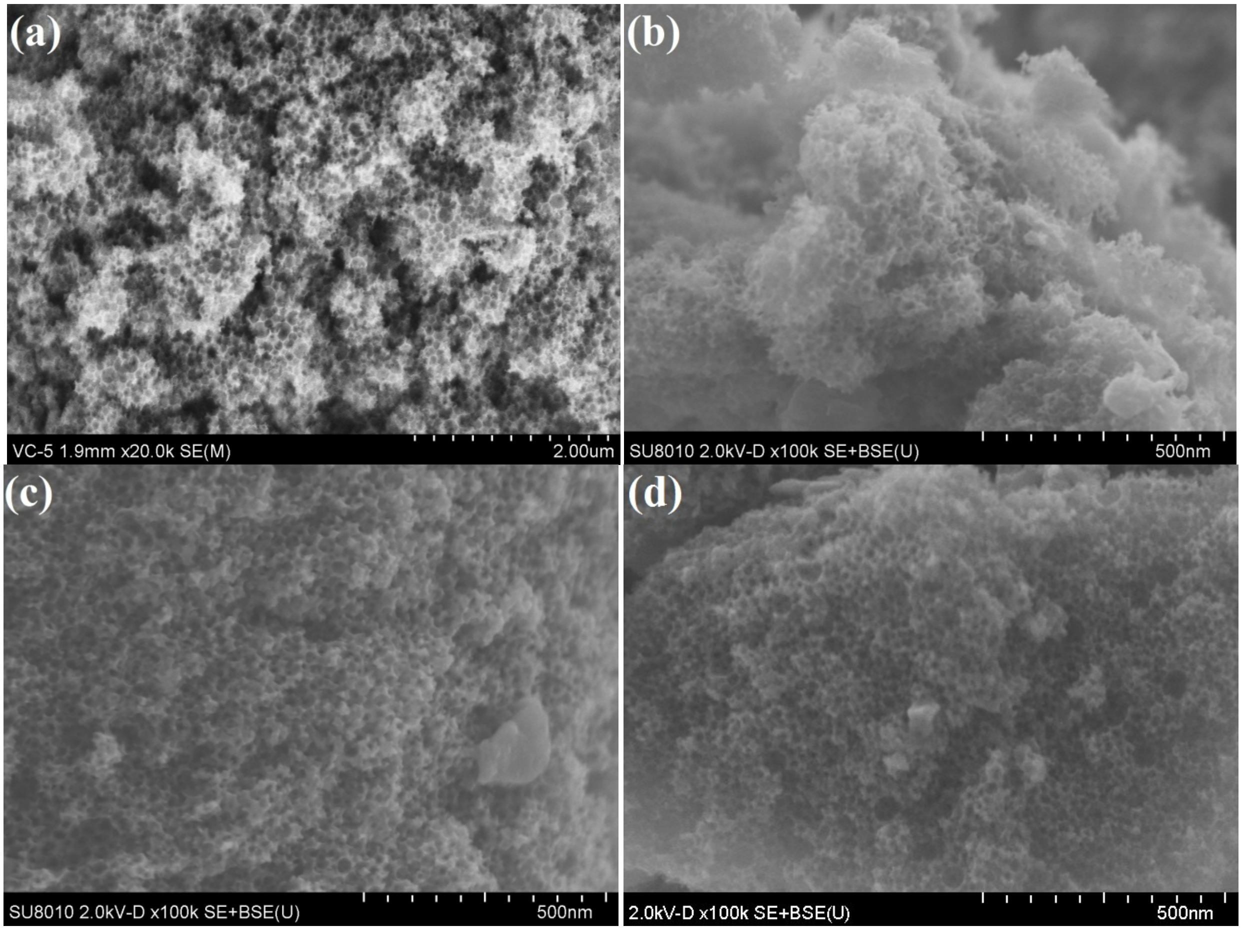

Figure S7. SEM images of CC (a), **CCO** (b), **CCONa (**c) and A**u(Salen)@CC** (d).

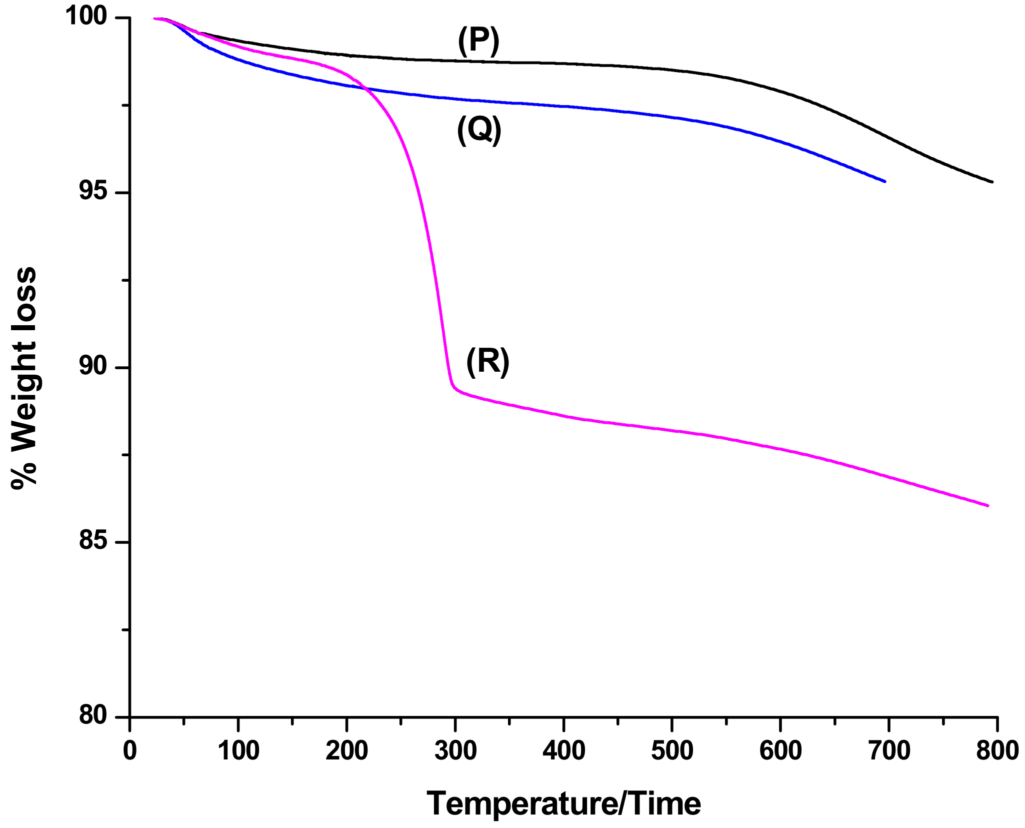

Figure S8. TGA curves of **CCO** (P), **CCONa** (Q) and **Au(Salen)@CC** (R).

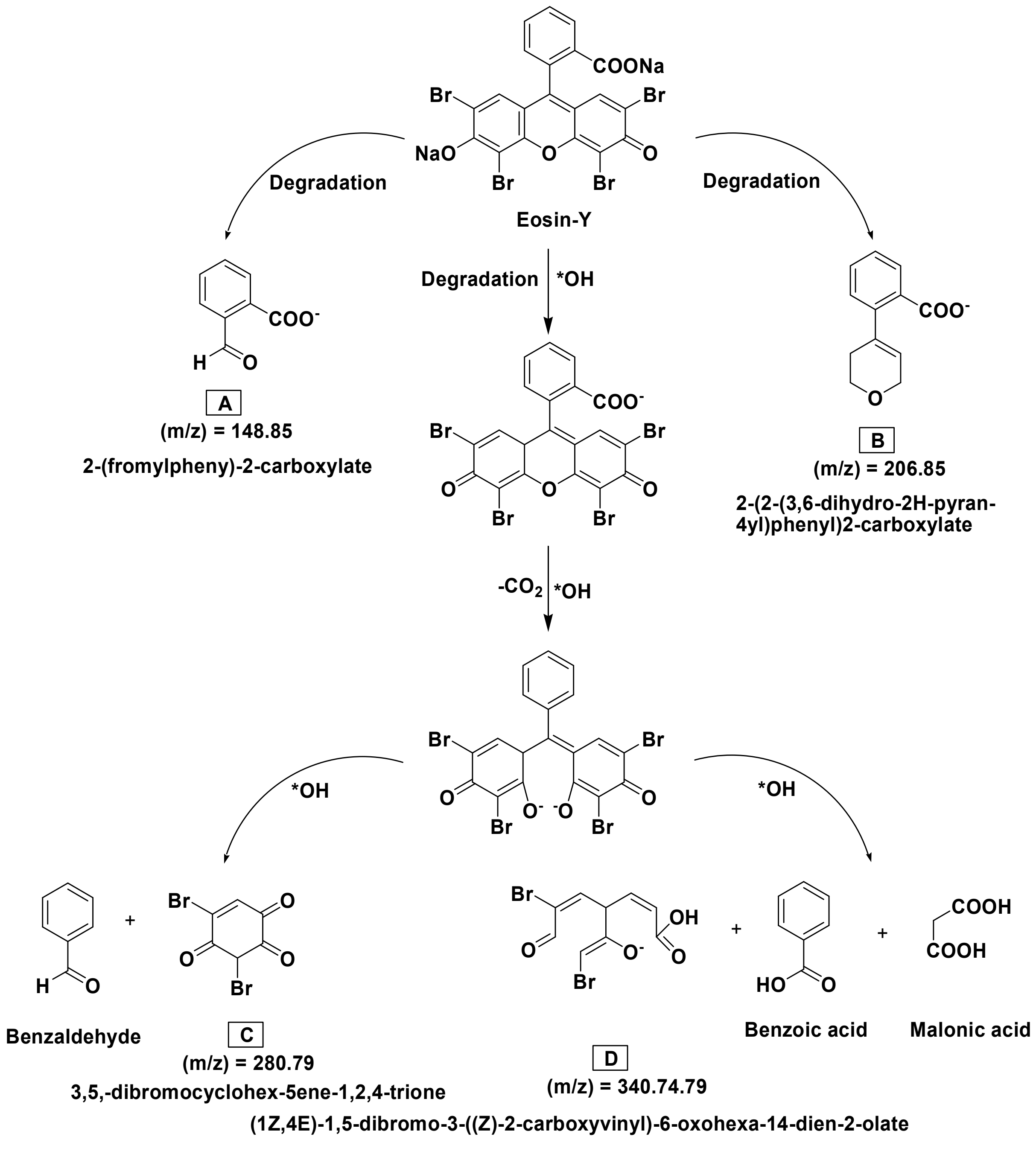

Figure S9. The possible degradation mechanism of Eosin-Y using **Au(Salen)@CC** catalyst.

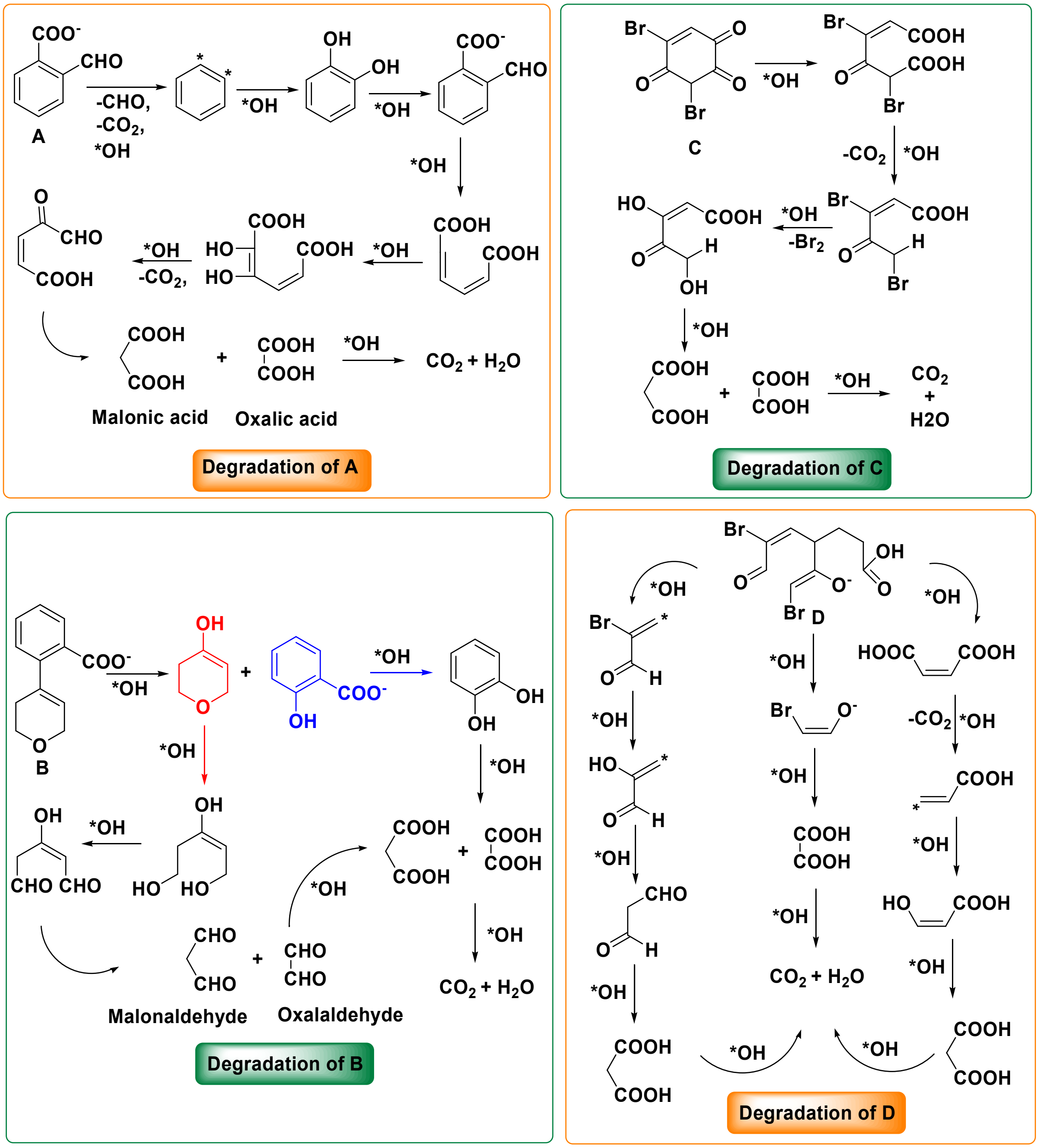

Figure S10. The possible degradation pathway of **A**, **B**, **C**, and **D** intermediate of Eosin-Y.

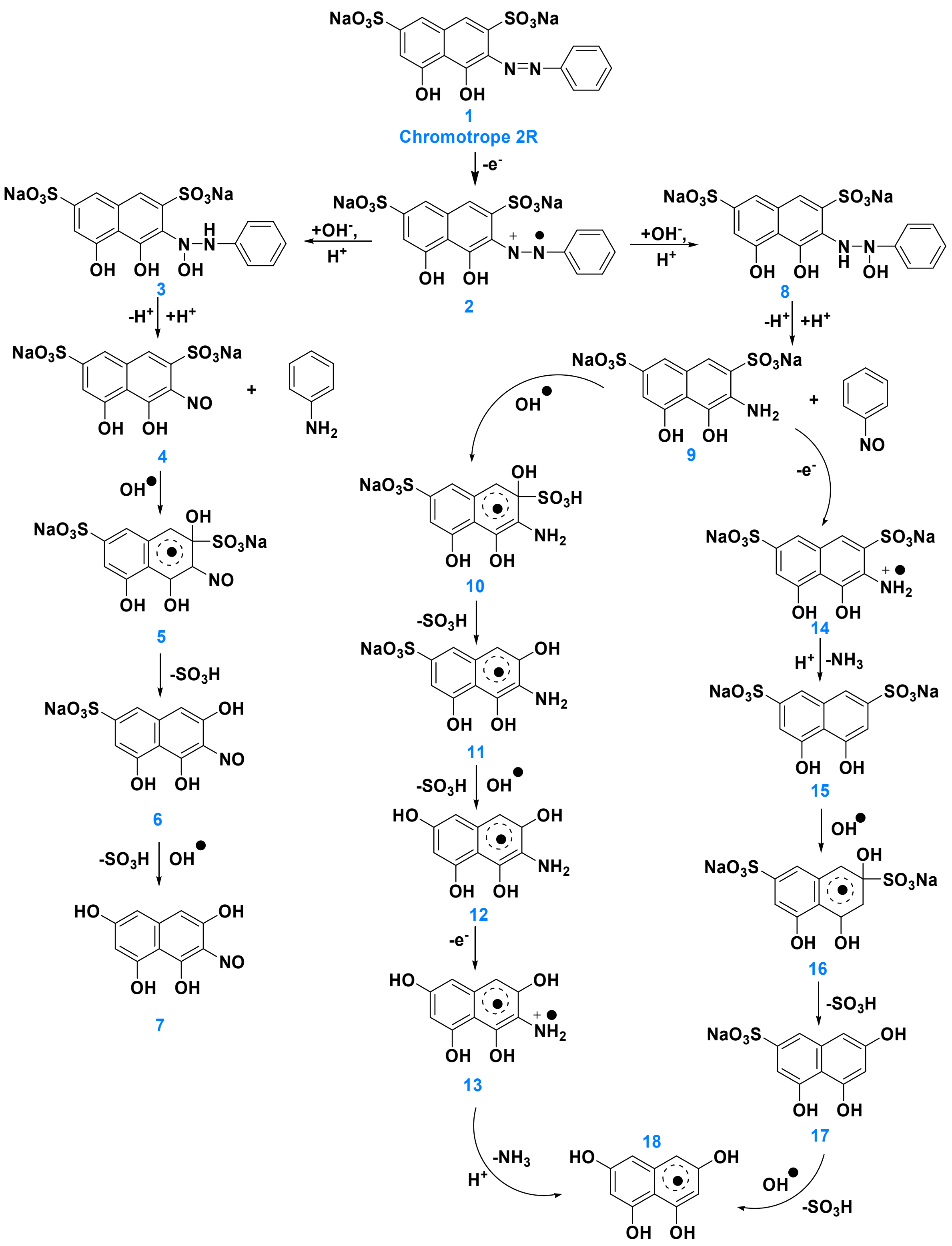

Figure S11. The possible degradation mechanism of Chromotrope 2R.

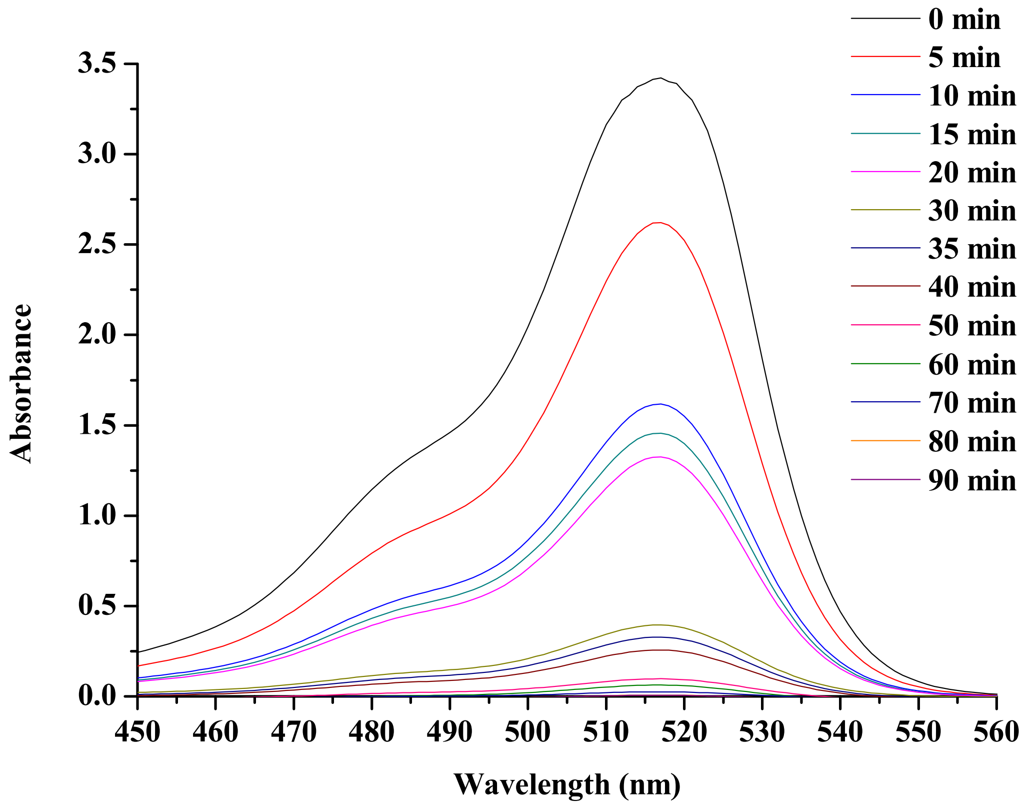

Figure S12. UV-vis absorption spectra of Eosin Y degradation with 1 g L^{-1} of **Au(Salen)@CC** catalyst load.

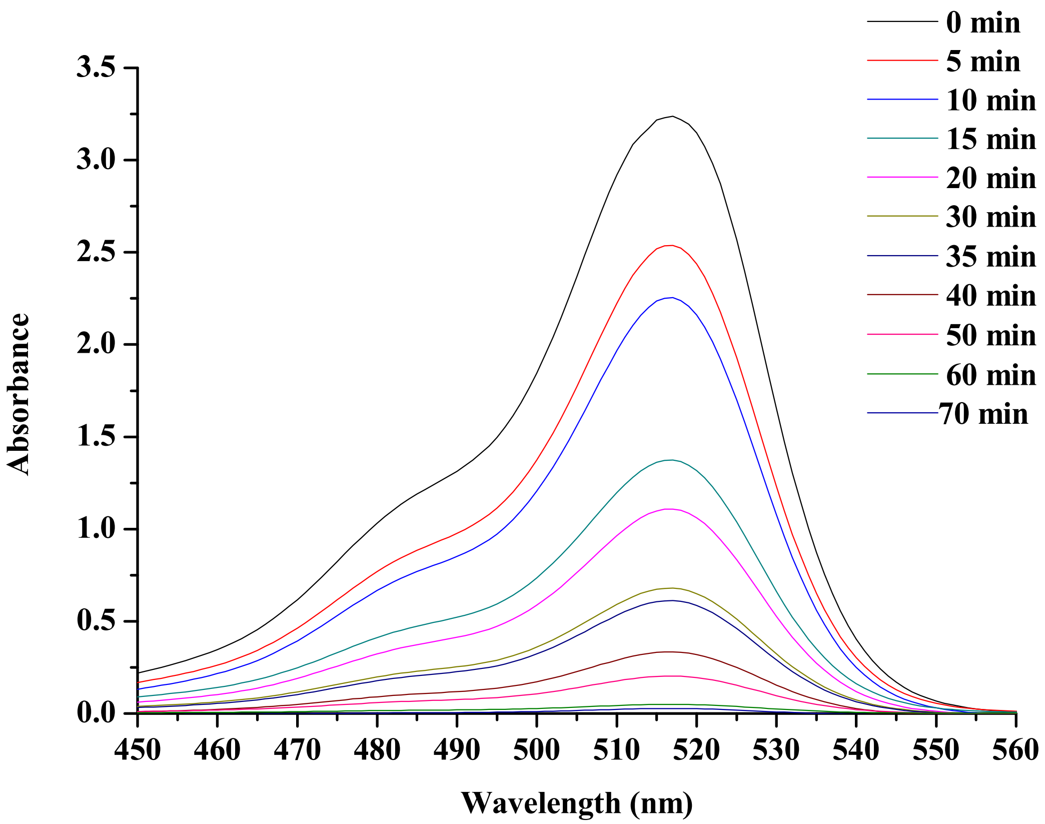

Figure S13. UV-vis absorption spectra of Eosin Y degradation with 3 g L^{-1} of **Au(Salen)@CC** catalyst load.

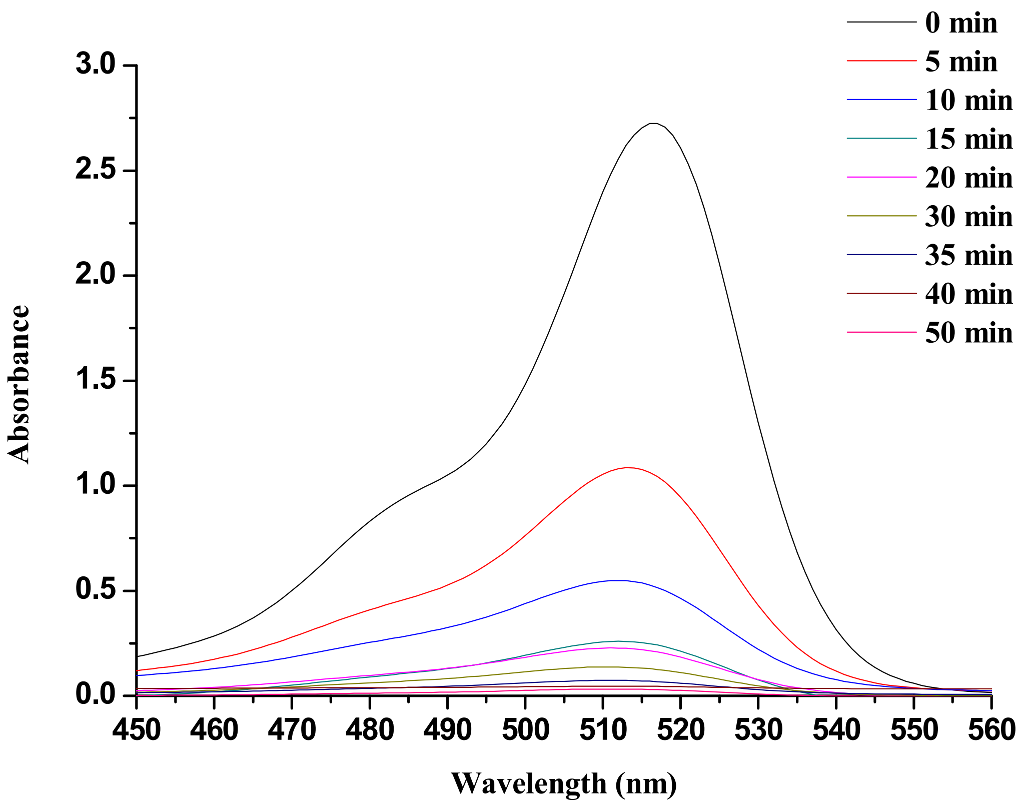

Figure S14. UV-vis absorption spectra of Eosin Y degradation with 5 g L^{-1} of **Au(Salen)@CC** catalyst load.

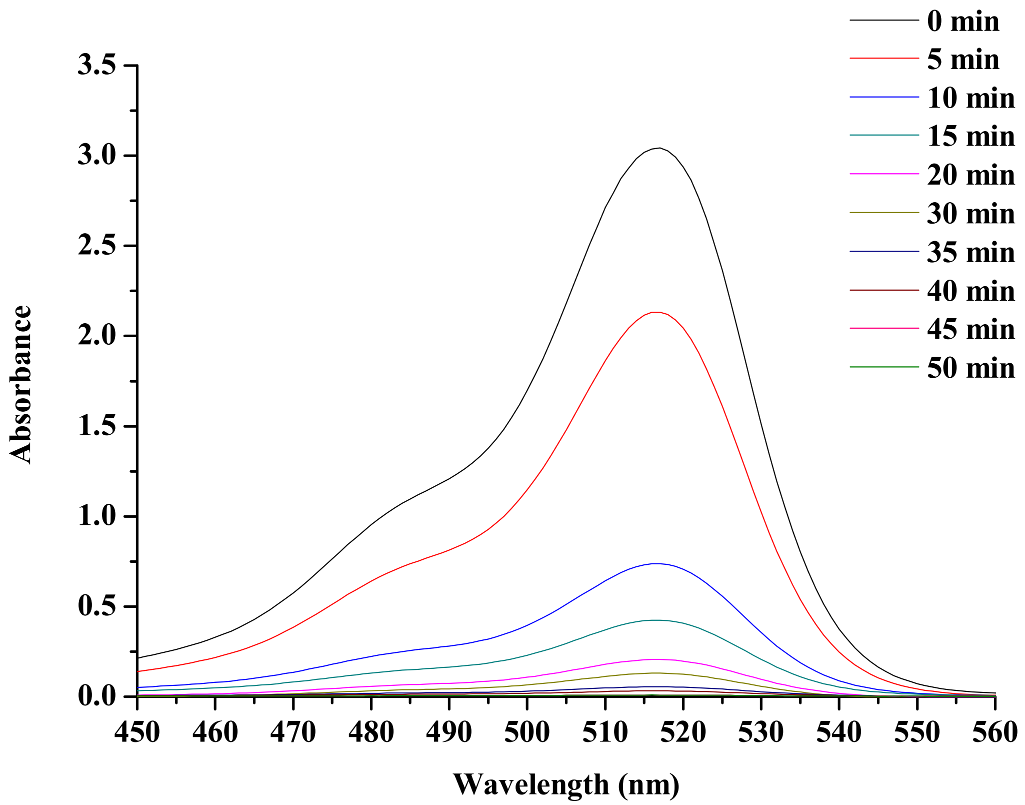

Figure S15. UV-vis absorption spectra of Eosin Y degradation with 8 g L^{-1} of **Au(Salen)@CC** catalyst load.

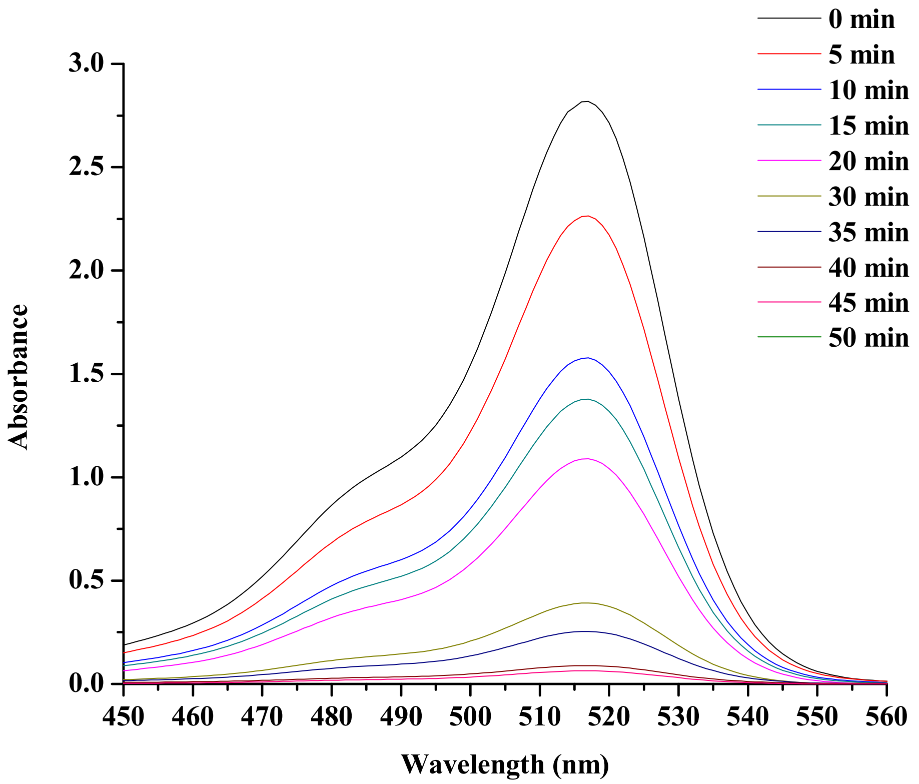

Figure S16. UV-vis absorption spectra of Eosin Y degradation with 10 g L^{-1} of **Au(Salen)@CC** catalyst load.

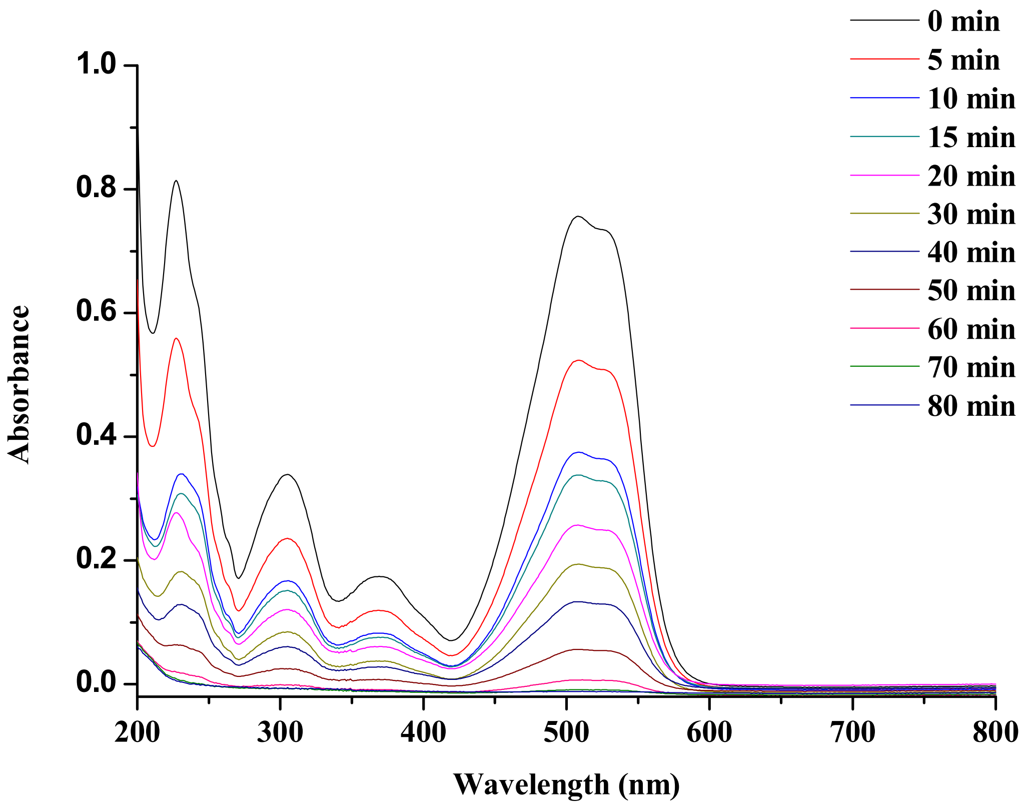

Figure S17. UV-vis absorption spectra of Chromotrope 2R degradation with 1 g L^{-1} of **Au(Salen)@CC** catalyst load.

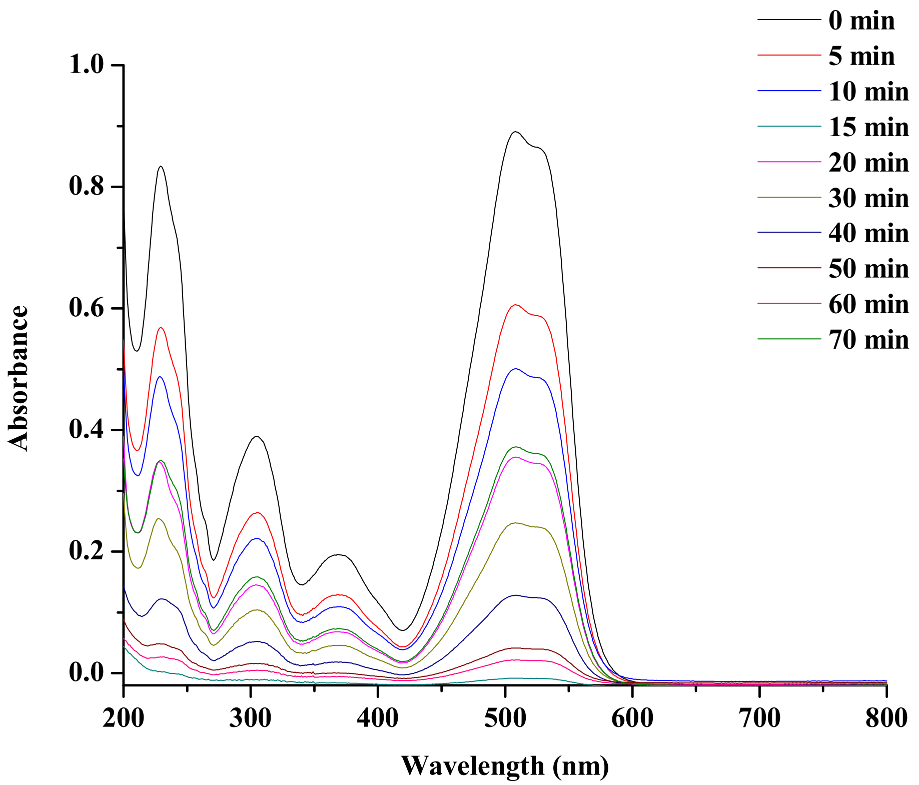

Figure S18. UV-vis absorption spectra of Chromotrope 2R degradation with 3 g L^{-1} of **Au(Salen)@CC** catalyst load.

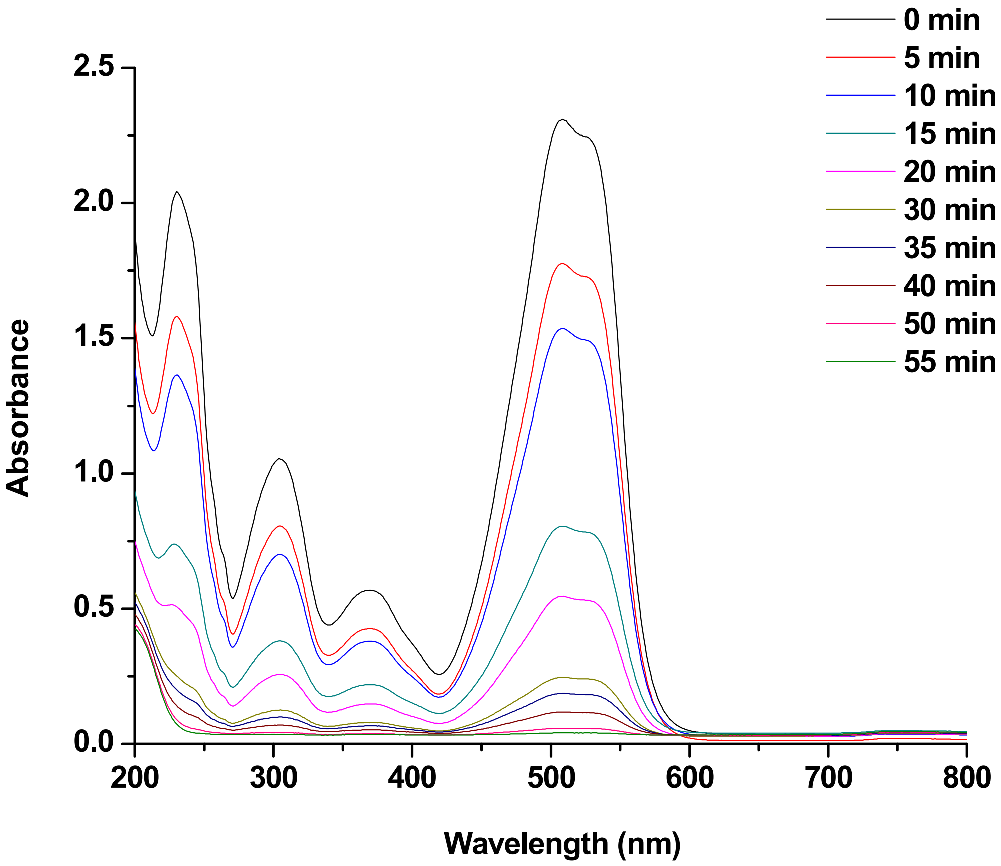

Figure S19. UV-vis absorption spectra of Chromotrope 2R degradation with 5 g L^{-1} of **Au(Salen)@CC** catalyst load.

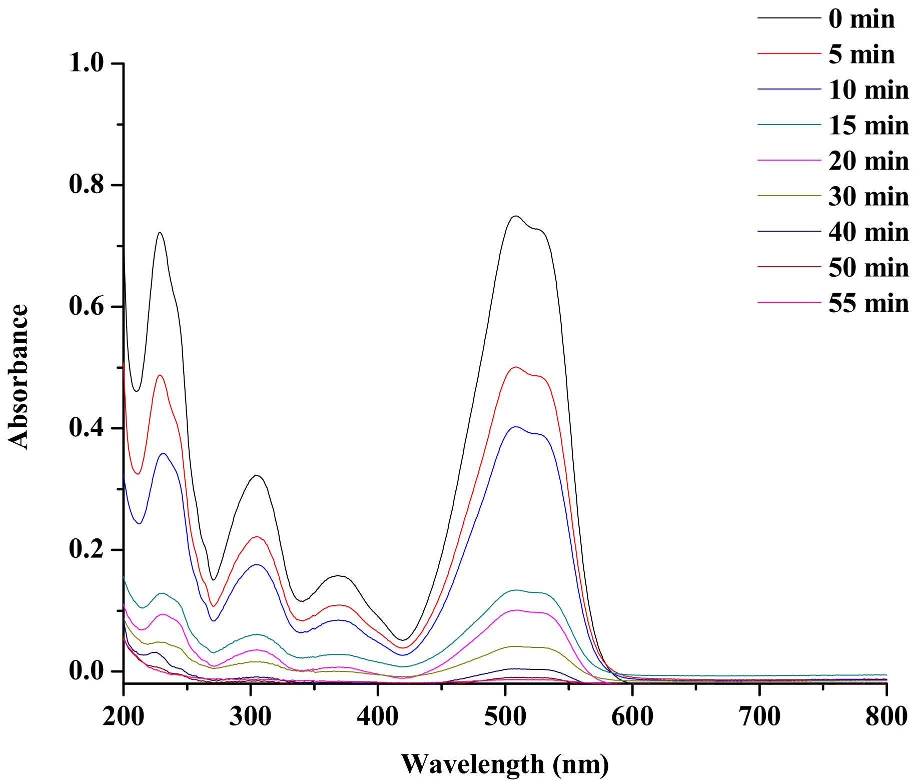

Figure S20. UV-vis absorption spectra of Chromotrope 2R degradation with 8 g L^{-1} of **Au(Salen)@CC** catalyst load.

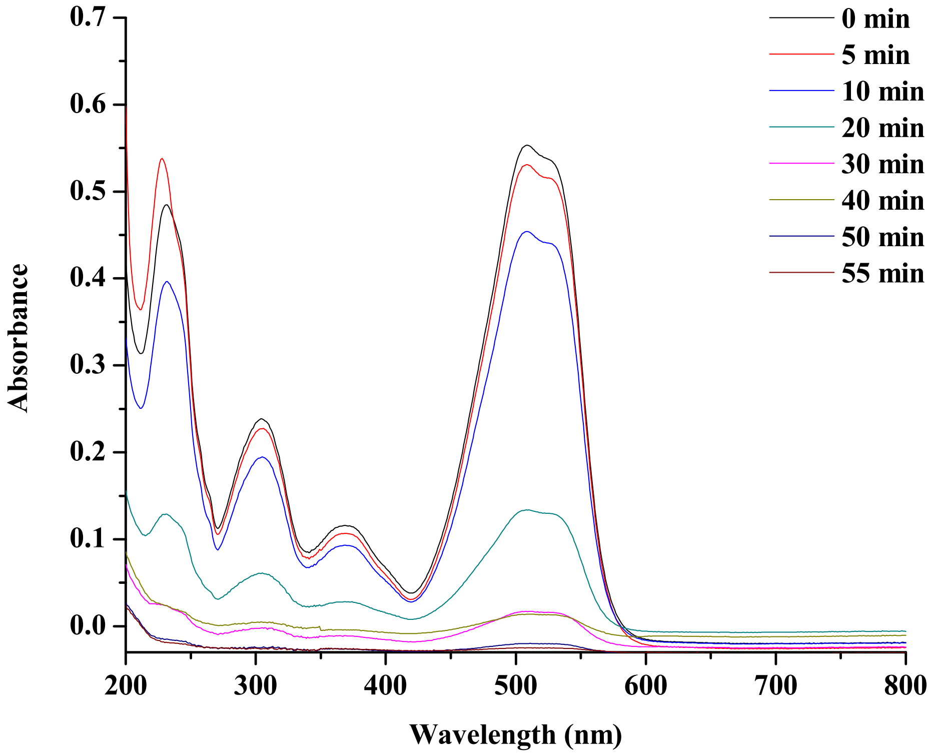

Figure S21. UV-vis absorption spectra of Chromotrope 2R degradation with 10 g L^{-1} of **Au(Salen)@CC** catalyst load.

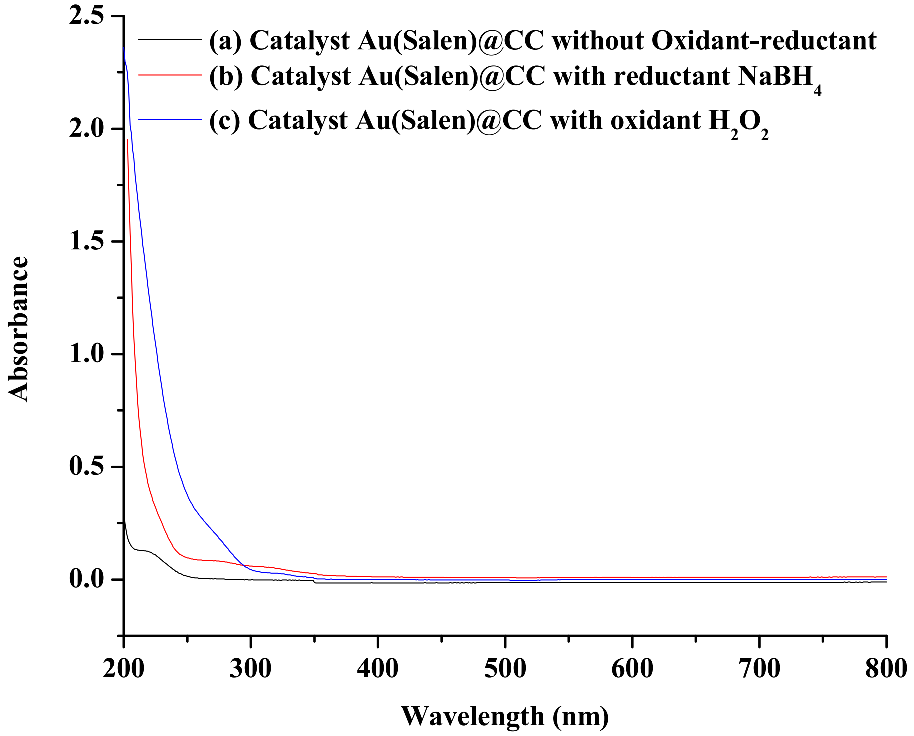

Figure S22. UV-vis absorption spectra of pure catalyst **Au(Salen)@CC** (a) without oxidant-reductant, (b) with reductant $NaBH_4$ and (c) with oxidant H_2O_2.

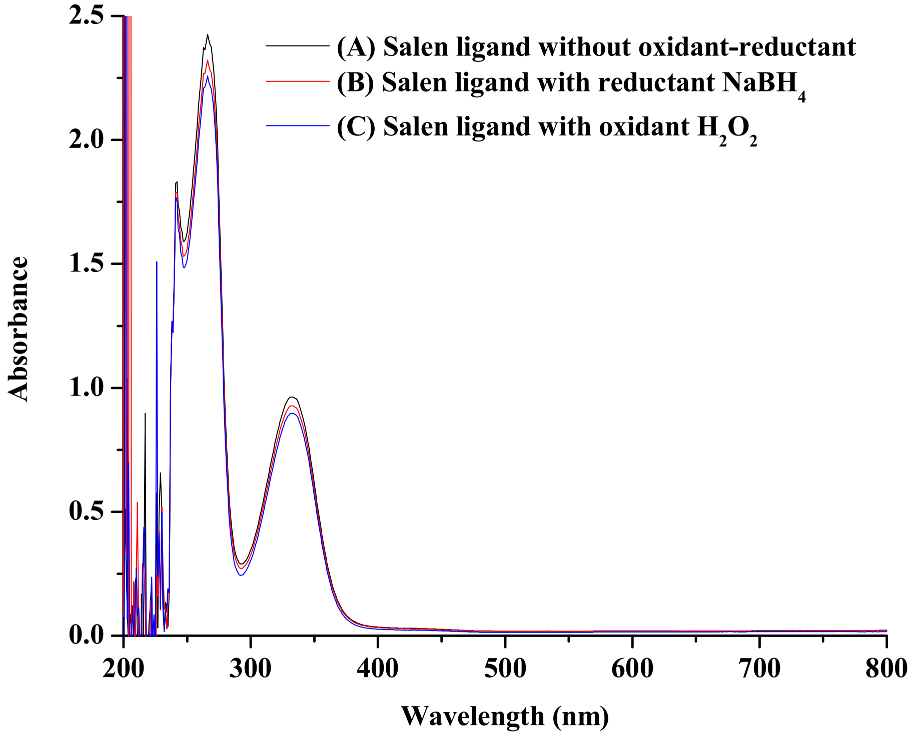

Figure S23. UV-vis absorption spectra of pure Salen ligand (A) without oxidant-reductant, (B) with reductant $NaBH_4$ and (C) with oxidant H_2O_2.

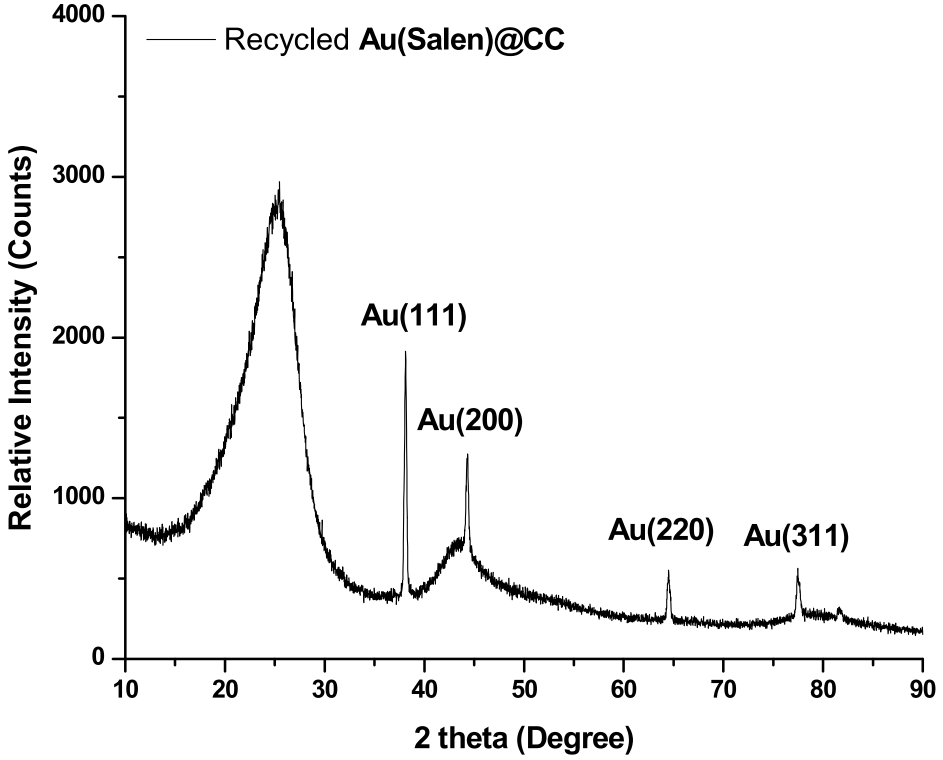

Figure S24. Wide-angle powder XRD patterns of recycled **Au(Salen)@CC** catalyst.
